# Enhanced handover mechanism using mobility prediction in wireless networks

**DOI:** 10.1371/journal.pone.0227982

**Published:** 2020-01-24

**Authors:** Khong-Lim Yap, Yung-Wey Chong, Weixia Liu

**Affiliations:** 1 National Advanced IPv6 Centre, Universiti Sains Malaysia, USM, Penang, Malaysia; 2 Internet Innovation Research Center, Minjiang University, Fuzhou, China; Wuhan University, CHINA

## Abstract

The rapid increase in the usage of the mobile internet has led to a great expansion of cellular data networks in order to provide better quality of service. However, the cost to expand the cellular network is high. One of the solutions to provide affordable wireless connectivity is the deployment of a WiFi access point to offload users’ data usage. Nevertheless, the frequent and inefficient handover process between the WiFi AP and cellular network, especially when the mobile device is on the go, may degrade the network performance. Mobile devices do not have the intelligence to select the optimal network to enhance the quality of service (QoS). This paper presents an enhanced handover mechanism using mobility prediction (eHMP) to assist mobile devices in the handover process so that users can experience seamless connectivity. eHMP is tested in two wireless architectures, homogeneous and heterogeneous networks. The network performance significantly improved when eHMP is used in a homogeneous network, where the network throughput increases by 106% and the rate of retransmission decreases by 85%. When eHMP is used in a heterogeneous network, the network throughput increases by 55% and the retransmission rate decreases by 75%. The findings presented in this paper reveal that mobility prediction coupled with the multipath protocol can improve the QoS for mobile devices. These results will contribute to a better understanding of how the network service provider can offload traffic to the WiFi network without experiencing performance degradation.

## Introduction

The growth in mobile and wireless networks has disrupted the way that networks were designed. A recent report showed that mobile data usage grew 63% in 2016 and that it will surpass 49 exabytes by 2021 [1]. Smart phones have eclipsed personal computers, and the global population of over 7.4 billion wireless users continues to consume an increasing amount of spectrum resources. Cloud-based services and video traffic as well as essential online services such as e-banking, e-learning and e-health continue to proliferate, causing the wireless traffic volume to grow exponentially.

Users’ expectations also change, and the demand for “anytime, anywhere” connections has become a necessity to satisfy their needs. The good news is that mobile devices are armed with multiple radio capabilities. However, current mobile devices do not have the intelligence to communicate via multiple wireless networks at the same time for improved capacity, coverage and seamless handover. There are no intelligent and autonomous mobile devices that are able to find and connect to the best radio networks that meet users’ needs. Users continue to experience intermittent connectivity and inconsistent throughput, and latency can be extremely unpredictable even when multiple radio networks are available. Since mobile devices cannot predict and optimize the handover process, the quality of service (QoS) for mobile users degrades further, especially when mobile users are on the move.

To improve the QoS, cellular service providers are integrating picocell networks to offer an economically appealing way to improve the coverage, QoS and network capacity. In addition, WiFi is used to offload the cellular network to create a heterogeneous network, allowing users to enjoy internet connectivity everywhere and at anytime. The emergence of WiFi has reduced the need to depend on the cellular network, especially when users are constantly moving inside a building. The provisioning of QoS has become more challenging due to the frequent and unnecessary handover process, either between WiFi and WiFi or WiFi and the cellular network.

The mobile handover process can be divided into three (3) stages, namely, handover initiation, network selection and handover execution [2]. Existing mobile devices will initiate the handover process when the connectivity between the mobile device and the connected access point (AP) or base station (BS) drops below a certain threshold of the received signal strength indicator (RSSI) level. The device will then select a network based on the RSSI because it is the simplest measurement even when the throughput is not optimal. This approach often causes data service session interruption and decreases users’ experience. Currently, mobile devices do not have the capability to connect to an optimal WiFi AP for a better QoS. This issue can indirectly cause unnecessary handover between different WiFi APs, eventually leading to service disruption whenever handover occurs. Unnecessary handover is also caused by the dense deployment of WiFi APs to cover a broad area.

One of the commonly used approaches to mitigate the frequent handover issue is mobility prediction. [3] proposed a mobility prediction model based on the hidden Markov model (HMM) to predict the next service eNodeB in the long-term evolution (LTE) cellular network. Mobility prediction-based approaches show promising solutions mainly because human mobility behaviour is far from random and is highly influenced by their historical behaviour. Although mobility prediction can determine the future location of users, improve the handover process, and manage the resources efficiently [4], the predictions only focus on the homogeneous network. In addition, the proposed algorithm has not been implemented to prove the concept of mobility prediction in helping the handover process, especially for both homogeneous networks and heterogeneous networks.

In a heterogeneous network, various network selection schemes and handover processes have been proposed to improve the QoS. One of the solutions takes advantage of the multipath scheme in order to efficiently and intelligently route the traffic to achieve a better QoS using the multipath transmission control protocol (MPTCP). MPTCP calculates the round trip time (RTT) and congestion windows of available network interfaces when deciding the network interface to be used. This protocol discovers the number of available paths available to users, establishes the paths and distributes traffic across these paths through creation of separate subflows based on the lowest RTT and unfilled congestion window. This approach in determining the best performing network interface by only considering the RTT may not be able to meet the requirement of the necessary high bandwidth, as the outgoing TCP request client packet is lightweight compared to the real packet containing content. Thus, the data rate of the lowest RTT network interface may not be capable of handling the required request.

There are three modes in MPTCP, namely, the full-MPTCP, backup and single-path mode proposed by [5]. Full-MPTCP mode introduces the usage of all subflows for communications from the device. Meanwhile, in the single-path mode, a new TCP subflow will only be established when the network interface breaks down. Unlike the single-path mode, the backup mode opens TCP subflows over all interfaces but only use one interface. When the main interface is down, the next network interface will be active to maintain the ongoing network session. MPTCP is designed such that it will “break-before-make”. When the single-path mode is active, the network interface will be used as long as it does not break down even when other subflows are better. Thus, [6] proposed a mechanism to cope with this situation by using network information such as the link quality of the network interface, which is retrieved from the system to determine the best network interface to use in the single-path mode [5]. However, link quality alone is not sufficient to be the deciding factor of the best performing network interface because system link quality information is merely calculated by the hardware itself based on the signal strength and radio frequency interference. This information cannot guarantee good network throughput and low packet loss to the user.

A study by [7] compared the performance between progressive mobility prediction using HMM and MPTP. When prediction was used, the number of handovers was reduced. This paper is an extension that integrates progressive mobility prediction using both HMM and MPTP. The paper is organized into five (5) sections. Section II presents the literature review. Section III gives an overview of eHMP, and section IV presents the performance of eHMP in heterogeneous and homogeneous networks. Section V summarizes the work of this research project.

## Related work

### Mobile IP

To sustain or improve the QoS in wireless networks, several protocols have been proposed for different layers. Mobile internet protocol (MIP) is one of the earliest solutions to ensure service sustainability in mobile devices. [8], which is a standard communication protocol conceived by the Internet Engineering Task Force standardized protocol, enables data usage to remain uninterrupted during mobile user device movement from one place to another.

**Fig 1:**
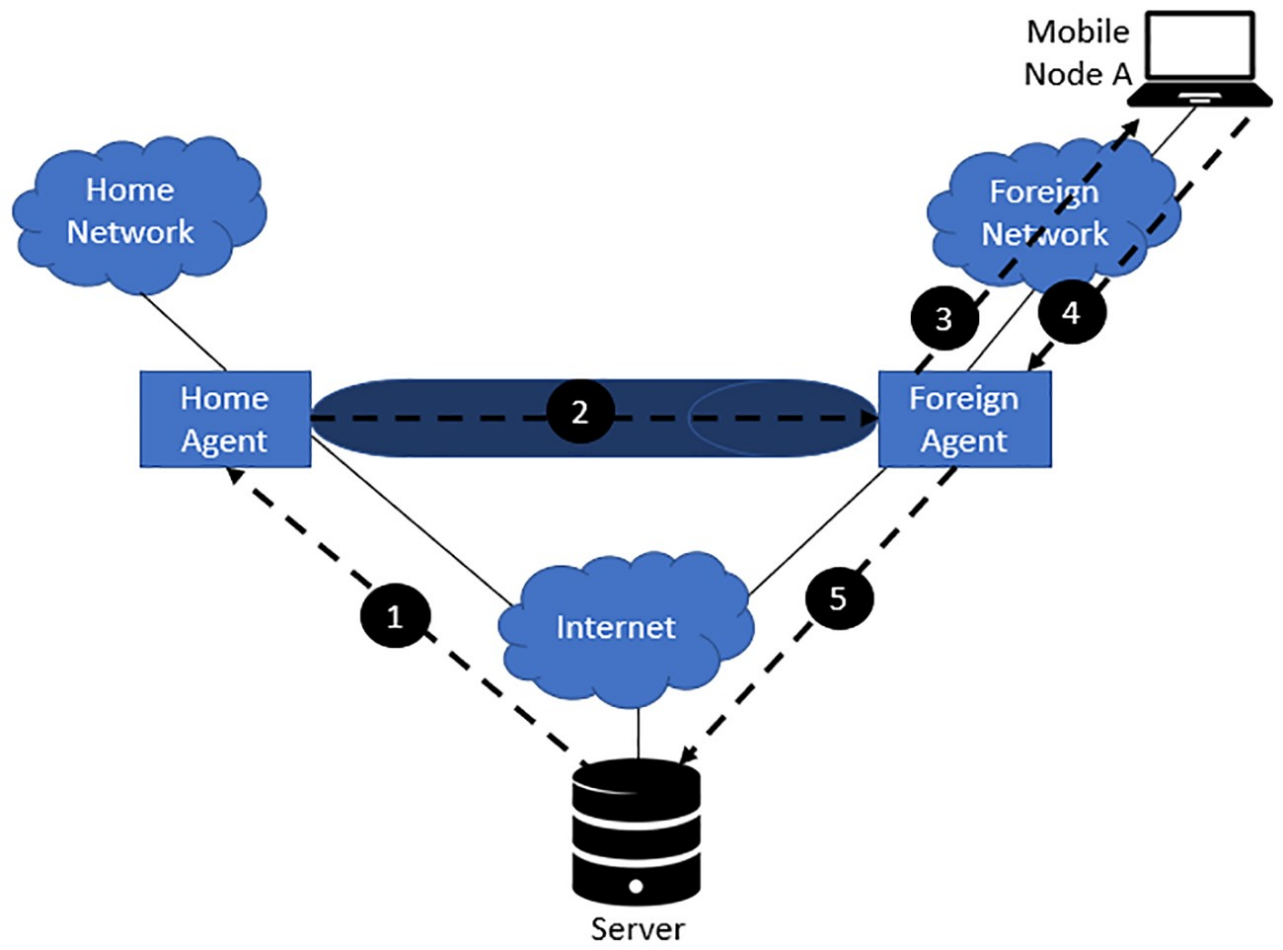
Triangle routing in mobile IP.

Mobile IP requires an HA to tunnel packets to a mobile device’s care-of address (CoA) whenever the mobile device leaves the home agent (HA). A CoA is assigned to a mobile device when a mobile device leaves the HA, and the address will be mapped with the home-of address (HoA) of the mobile device in order to locate the mobile device. However, triangle routing might occur because all packets from the corresponding node (CN) have to pass the HA to reach the mobile device despite the fact that the mobile device is just next to the CN, which incurs a heavy load on the HA and a waste of resources. Fig 1 shows an MIP scenario where a mobile device has moved from the home network to a foreign network.

As shown in Fig 1, there is a total of five (5) steps involved that indirectly cause triangle routing in MIP.

1. The server transmits an IP datagram with the destination of mobile device A, where the internet protocol (IP) header will hold A’s HoA. The datagram will then be routed to A’s home network.
2. Upon receiving the IP datagram destined for mobile device A at the home network, the home agent will encapsulate the entire datagram inside a new IP datagram, where the new header will have A’s CoA. Then, the datagram will be retransmitted again. The use of the additional IP datagram with a different IP address as described is also known as tunnelling.
3. The foreign agent removes the outer IP datagram header and encapsulates the original IP datagram in the network-level PDU and finally delivers the datagram to mobile device A across the foreign network.
4. If mobile device A wishes to reply and send the IP packet back to the server, the IP address that will be used as the destination in the packet will be the server’s address, as the server is a static node that has a fixed address in this example.
5. The IP datagram destined for the server will travel directly across the internet to reach the server using the server’s IP address.

Although MIP is still being used today, [9] have proposed a different transport mechanism incorporated with OpenFlow (OF) for routing and providing network connectivity to mobile devices. The proposed method succeeds in maintaining network connections under the same IP address even though the mobile device traverses to another location. It does not need a detour as in MIP that passes the HA, and as a result, flow entries can be constructed within OpenFlow switch (OFS) to update the new address of the mobile device whenever the node changes location. Meanwhile, the MIP controller is used to calculate the best path and install the corresponding flow entries in all of the required OFS. This approach is different from the conventional MIP, as the HA will be used to convert the destination’s IP as well as install flow entries along the chosen path until the packet can reach the last CoA. Meanwhile, the foreign switch’s role is to inform and update the controller for registering the mobile device upon mobile device movement from the home switch to the foreign switch.

Another approach proposed by [10] to alleviate or eliminate the triangle routing problem that exists in MIP uses the HA to identify each mobile device. The proposed approach allows the mobile device’s latest location represented by its CoA, the mobile device’s first-hop OpenFlow switch. Meanwhile, the OpenFlow controller’s role in this approach is to maintain a binding cache that can map a mobile device’s HoA to CoA. With this approach, the CN can reach the mobile device by sending data packets out from its switch to the mobile device HoA without taking a detour to the HA. However, if no single rule in any of the flow tables matches the destination IP address in an OFS, the packet-in message is sent to the controller to look for the mobile device CoA according to the mobile device HoA, and that flow table entry is installed on the CN switch so that all packets will have the destination address rewritten to be the mobile device CoA, the mobile device’s first-hop OpenFlow switch. Upon reaching the mobile device CoA, the packet will have the destination address rewritten to the mobile device HoA. The above process is done so that it is transparent to the end hosts. This approach is different from the conventional MIP, as the HA is not routable and the binding cache placement problem is also addressed, where the optimal forwarding path between the mobile device and CN is ensured instead of the triangle routing that might happen due to taking a detour to the HA.

Several updates have been made to MIPv6 so that foreign agents are no longer required; [11] proposed an OF-based Proxy MIPv6 over software-defined networking (SDN), a mechanism that supports flexible configuration architecture. This is done by separating mobility functions to the controller for the purpose of increasing capacity management and improving the resilience to failures. Moreover, the problem of IP-in-IP tunnelling overhead is mitigated by using flow table-based routing; therefore, routing is optimized, and the triangle routing issue that appeared in PMIP is solved. Furthermore, the result of the obtained performance evaluation showed that the proposed approach outperforms PMIPv6.

Although several approaches have been proposed to alleviate the issue from MIP and improve handover, these approaches have challenges in implementation. For instance, compared to the traditional implementation, all the works require the addition of new software controllers. This is a major challenge for operators, as the distance travelled by a mobile user is substantial, meaning that a new standardized protocol similar to MIP is required to govern the compatibility between network providers and service providers to maintain the functionality regardless of where the users go. Different service providers have different network vendors that utilize unique network functionality from different hardware vendors to provide different performance tiers to end users, hence collecting different subscription fees. Therefore, the association of enormous service providers for the purpose of utilizing new technology such as SDN for guaranteeing excellent network performance to mobile users requires a great effort from both government and top-level management.

### Multipath TCP

Transmission control protocol (TCP) is able to transfer packets reliably. However, the protocol restricts communications to a single path per transport connection [12], which is opposed to the opportunities offered currently where multiple paths such as WiFi and the cellular network exist between peers in mobile devices. The simultaneous use of these multiple paths for the TCP session would improve resource usage within the network, thus improving the user experience through higher throughput and improved resilience to network failure [13].

**Fig 2:**
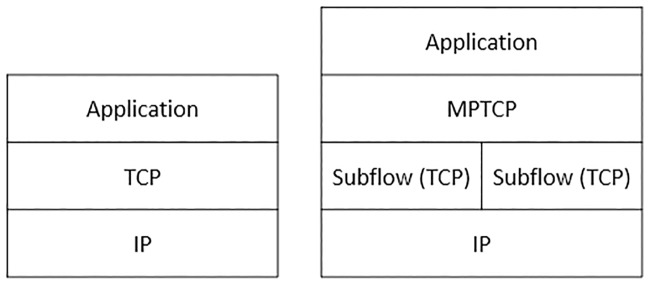
Standard TCP and MPTCP protocol stacks.

Multipath TCP (MPTCP) serves as an extension to TCP by providing the ability to use the multiple-path service, as shown in Fig 2. This approach enables a transport connection to operate across multiple paths between peers simultaneously. In addition, MPTCP is designed to be backwards-compatible with regular TCP in order to increase the chance of deployment. The protocol should be able to fall back to standard TCP with no extra actions from the user and communicate with legacy hosts. Hence, the first data packet exchange between peers decides if a standard TCP or MPTCP will be used. There are a total of six MPTCP operations that can happen in the MPTCP connection. The operations are the following:

1. MPTCP connection initiation.
2. Association of a new subflow with the existing MPTPCP connection.
3. Informing the potential address to other hosts.
4. Data transfer using MPTCP.
5. Changes in subflow priority.
6. Connection closes.

**Fig 3:**
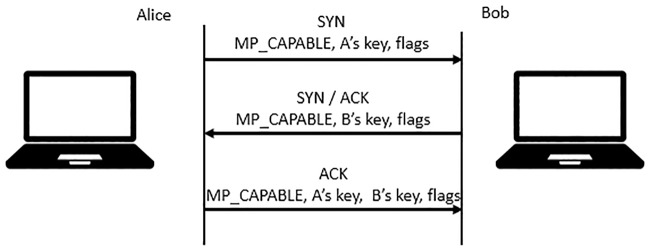
Initiating an MPTCP connection.

MPTCP uses the same signalling as that for initiating a normal TCP. However, the SYN, SYN/ACK and ACK packets carry an additional option, namely, “MP CAPABLE”. This option is used to verify if the remote host supports MPTCP and authenticates the establishment of additional subflows. Fig 3 shows the establishment of the MPTCP connection between Alice and Bob.

**Fig 4:**
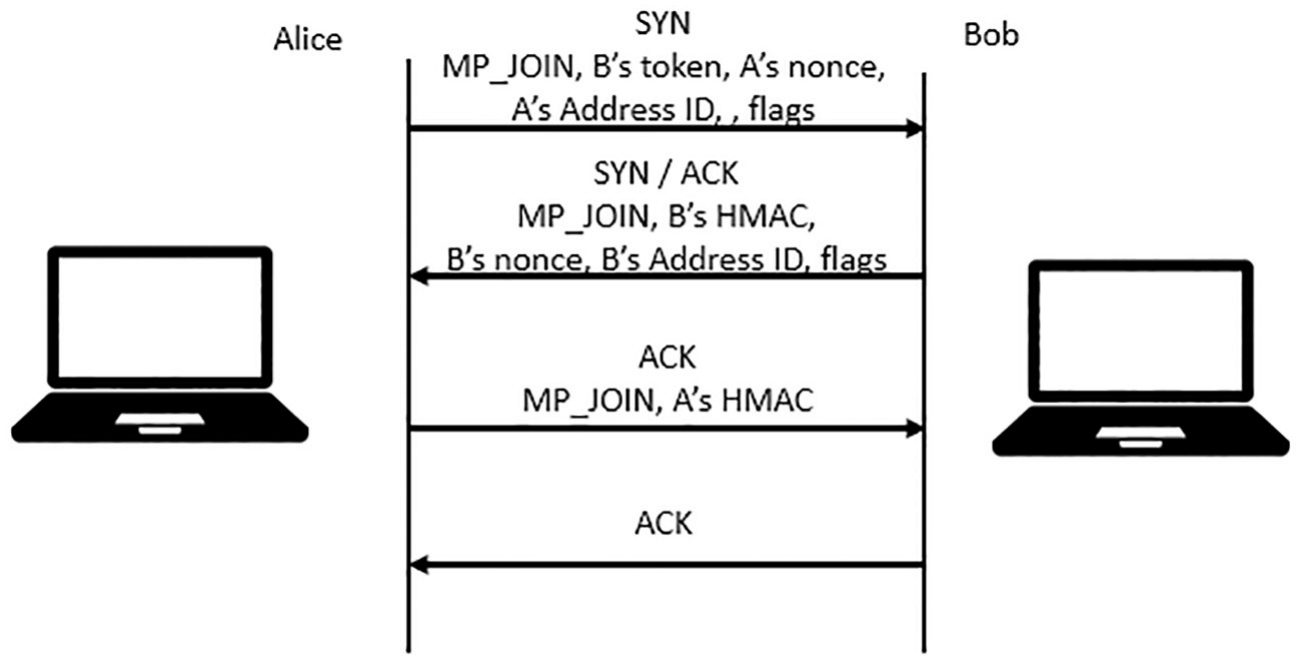
Starting new subflow.

Additional subflows can be added to the MPTCP connection with “MP JOIN” once the MPTCP connection has begun with the “MP CAPABLE” exchange. Similar to the previous connection initiation, the new subflow started as the normal TCP SYN/ACK exchange. In addition, the join connection (“MP JOIN”) TCP option is used to identify that the new connection will be joined by the new subflow. Moreover, the key exchanged previously will now be used to generate a token that is used to identify which MPTCP connection it is joining, and the hash-based message authentication is used for authentication. A random number (nonces) is also exchanged between hosts to prevent replay attacks on the authentication method. The address ID, as shown in Fig 4, identifies by the hosts themselves which local address is mapped to the address ID.

**Fig 5:**
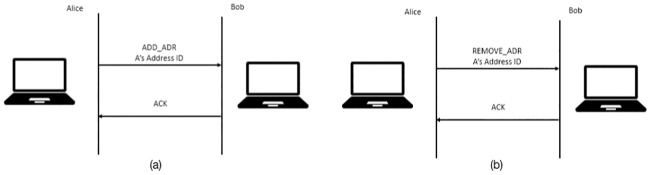
(a) Address addition, (b) Address removal.

The host with multiple IP addresses may change during the lifetime of an MPTCP connection. The addition of the new subflow for multiple addresses is described above. However, a host may only want to advertise the availability of an address without establishing a new subflow to a remote host in certain circumstances. In this situation, the “ADD ADDR” TCP option together with the address ID is used to inform the remote host about the alternative IP address without establishing a new subflow. Meanwhile, if a previously advertised address becomes invalid, the host should announce this message to the remote host in order for both peers to remove the address or subflow related to this address. This can be achieved through the TCP option “REMOVE ADDR” together with the address ID that was used previously mapped with the invalid IP address. Fig 5 shows how the options “ADD ADDR” and “REMOVE ADDR” are used.

**Fig 6:**
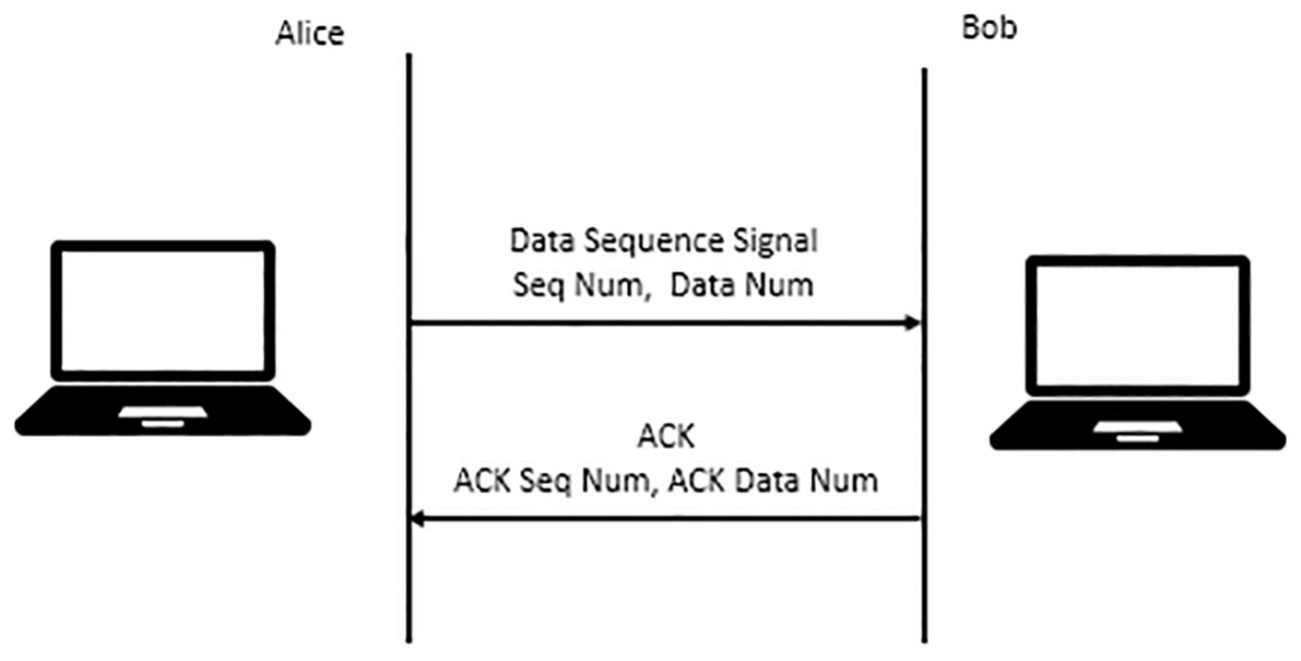
Transfer of data using MPTCP.

Since MPTCP supports transferring data over multiple TCP subflows, the reliability of data delivery is important to ensure in-order delivery. The data sequence signal (DSS) option is used for data sequence mapping and data ACK. In particular, MPTCP uses a 64-bit data sequence number to number all data sent over MPTCP connection. Each subflow has a 32-bit subflow sequence number space, and MPTCP uses this subflow sequence space to the data sequence space; thus, data can be retransmitted on different subflows in the event of any failure. Meanwhile, data ACK acknowledges receipt of data at the connection level. Fig 6 shows the illustration of MPTCP data transfer.

**Fig 7:**
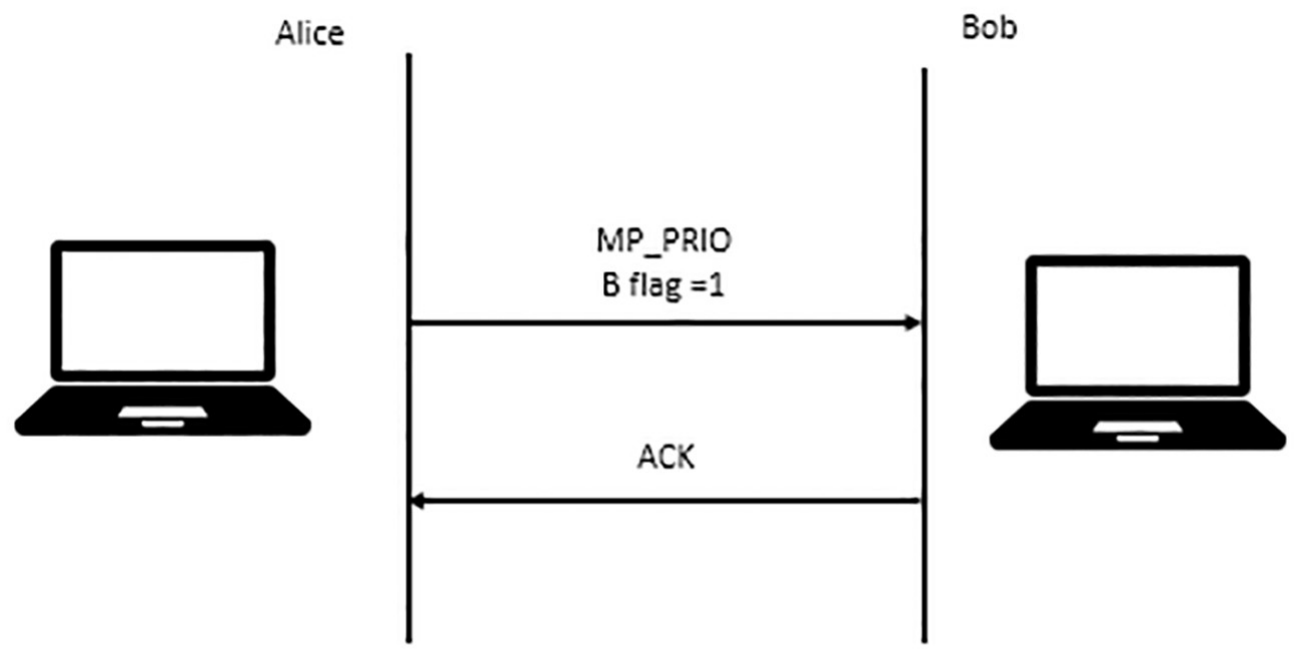
Change in MPTCP subflow’s priority.

The usage of multiple subflows in MPTCP provides advantages of combined throughput and added resilience to the connection failure compared to that of traditional MPTCP. In addition, should a host wish to decide how traffic is sent over available paths, option “MP PRIO” can be used to signal a change in the priority of the subflow. There are two options available to be set, namely, the regular and backup path. The regular path subflow will be used to transfer data as usual, while backup subflow will only be used if there are no paths available. Fig 7 shows how host Alice configures a subflow as a backup path by using the “MP PRIO” option with the “B” flag set to 1.

**Fig 8:**
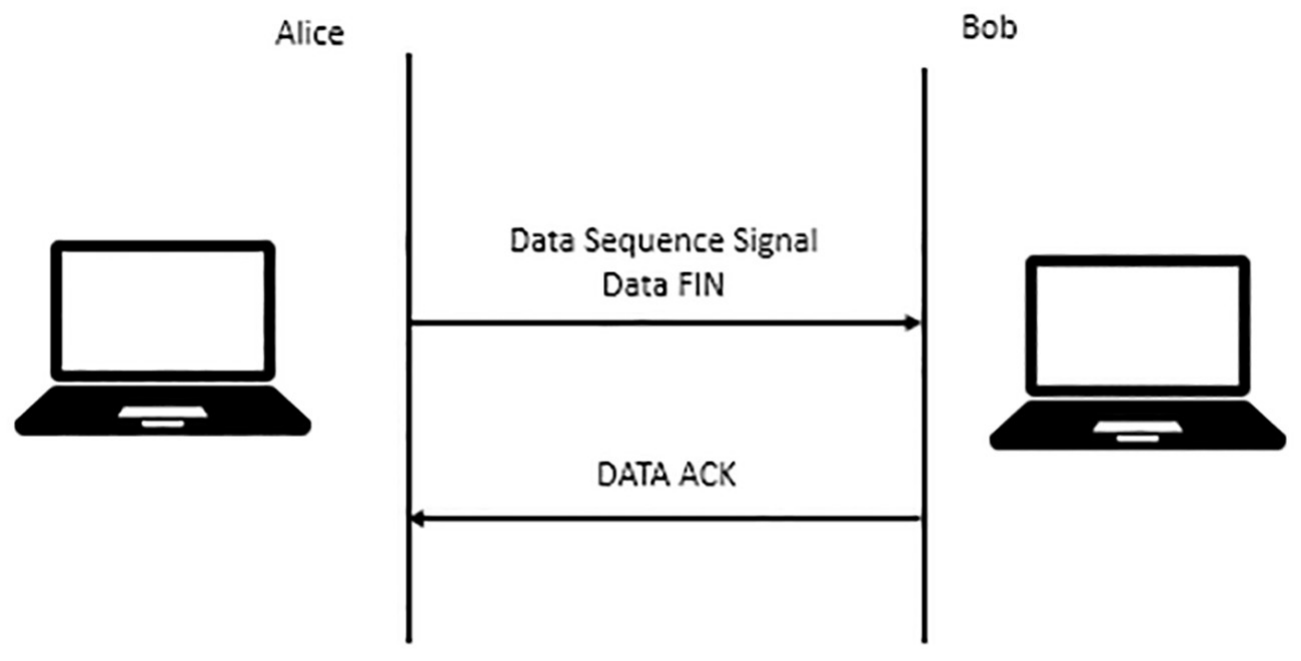
The closing of the MPTCP connection.

Upon completion of sending data to the remote host, “Data FIN” is sent as part of the DSS to signal no more data to be sent. This behaviour acts similar to a regular TCP FIN. Once the remote host determines that all data have been successfully received, “DATA ACK” will be used to acknowledge the completion of the MPTCP connection. Fig 8 illustrates how the MPTCP connection will be closed by using the DSS option.

### Mobility prediction

The rapidly increasing demands of the mobile internet have led to a wide deployment of the 3G/4G cellular network, which offers good coverage and bandwidth. Nevertheless, the challenge of a high implementation cost is faced by operators and mobile users. In particular, a spectrum license is required in order to operate the cellular network, and end users are required to pay a subscription fee to enjoy cellular data network capability.

Compared to cellular data networks, WiFi is becoming the preferred solution due to its cost and availability. Hence, WiFi access points (APs) are widely deployed for mobile users to enjoy free access to the internet. Nevertheless, concerns such as the frequent and unnecessary handover between different WiFi APs starts to worry mobile users due to the high power consumption and intermittent performance. One reason this issue happens is due to dense deployment of WiFi APs to cover a broad area. Moreover, the nature of mobile users, in that they are constantly moving from one location to another, means that they will eventually encounter a service disruption whenever handover occurs. Thus, mobility prediction is one of the regularly used approaches to predict the future location of mobile users in order to provide more efficient resource and handover management.

Considering the benefit of mobility prediction, [14] proposed an efficient handover mechanism that reduces the ping-pong effect and maximizes the residence time in a target femtocell. The hidden Markov model (HMM) is used as the prediction scheme, which is based on current and historical movement information of user equipment (UE) as well as signal quality of the femtocell AP to predict the next location of the next assigned femtocell AP. The proposed approach successfully minimizes the number of handovers where mobile users stay connected to the same femtocell AP for a longer duration. Since frequent handovers of moving mobile devices may affect the throughput of the device, [15] proposed association control policies to better understand the relationship of handover frequency, achievable throughput and mobility prediction of mobile devices in the dense femtocell.

[3] utilize mobility prediction to predict the next service eNodeB used in the long-term evolution (LTE) network to improve communication. The proposed approach has used the trajectories’ characteristics of eNodeB accessed as the hidden state and the individual’s historical service eNodeB as the observable state in the HMM model. By experimenting with real-world cellular network data, the results showed that the model exploits more knowledge on the user mobility behaviour with a higher number of HMM hidden states. Nevertheless, this approach may lead to additional invalid sequences and computing complexity. HMM learning may be impacted by insufficient length of training sequences due to the lack of information, resulting in high invalid prediction and low accuracy. The length of the observable state, which is also known as users’ historical paths, plays an important role in the prediction performance. The results showed that nine (9) is the optimal length for user mobility pattern determination.

[16] also proposed a vertical handover method based on a prediction mechanism by using the method of the twice moving average. Aiming at solving the power consumption, the proposed vertical handover algorithm reduces the number of measurements of the existing vertical handover algorithm and takes the network conditions such as the received signal strength (RSS), bandwidth, signal delay, security and capability of the mobile device, such as power consumption, into consideration. By predicting the trend of the cost function and the RSS ahead of the phase of the handover discovery, only when the cost function is lower than zero will a handover by a mobile device be triggered. This action reduces the power consumption and selects the most optimal network. Similarly, [17] proposed two handover prediction schemes for LTE-WLAN heterogeneous networks. The first scheme depends on the quality of all signals among mobile stations, and the second scheme is a multi-criteria prediction based on the SNR value and the stations’ bandwidth. Both schemes achieve a success rate of 99% in the predefined simulation area.

Aside from using mobility prediction to predict a better network or a better femtocell to handover, spatio-temporal prediction and next-place prediction based on mobility prediction are proposed by [18]. The authors investigated the effect of living habits on the proposed prediction models and selected one from these two models for an individual to achieve effective mobility prediction at the users’ point of interest (POI). By clustering adjacent locations and identification of POI, users are grouped based on living habits during different time periods with user profiles. Then, spatio-temporal prediction is used to predict where a user would appear at a specific time in the future based on the correlation between time and places, and the next-place prediction is used to predict the next place that a user would visit after leaving the current place based on movement patterns. The results show that users’ entropy profiles affect the accuracy of spatio-temporal mobility prediction, where users with regular lives and with short movement patterns are better modelled for spatio-temporal prediction. Moreover, next-place prediction is suitable to model user mobility for those leading highly mobile lives.

### Hidden Markov model

**Fig 9:**
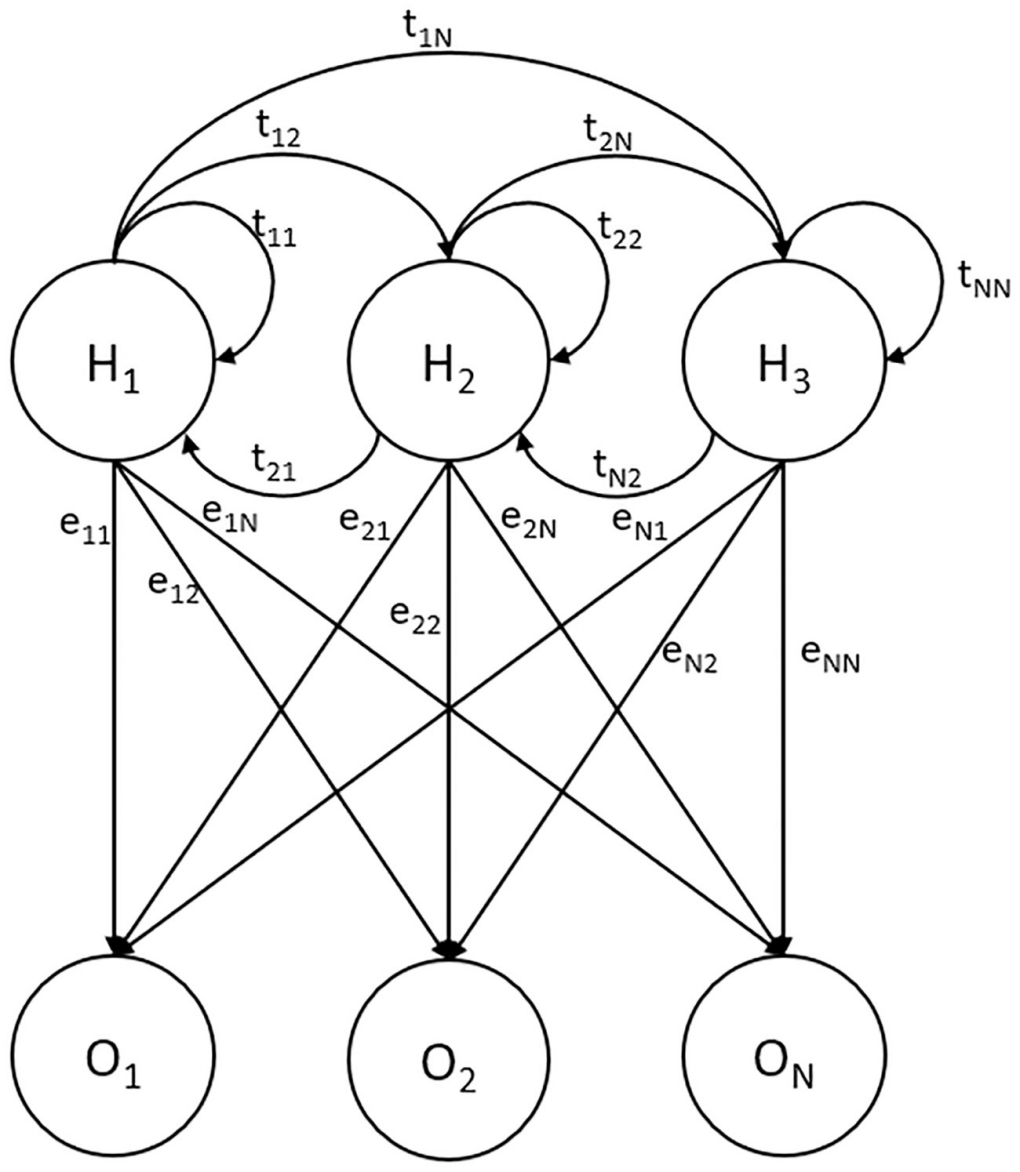
Hidden Markov model.

Two well-known mobility prediction-based approaches to predict the future location for mobile users are the HMM and the neural network. An HMM [19] consists of a finite set of states, namely, hidden states, a finite set of transition probabilities, a sequence of emission variables or output symbols, which are also known as observable states, and a set of emission probabilities. Transition probabilities denote the probability of a hidden state transition to another hidden state, whereas emission probabilities represent the distribution of output symbols that are emitted from each hidden state. Moreover, HMM is known as a doubly stochastic process with an underlying stochastic process that is not observable unless observed through another set of stochastic processes that produces the sequence of observable symbols [20], as shown in Fig 9.

An HMM can be defined as follows.

- H = H1 H2 … HN, H is the N hidden states of the system.
- O = O1 O2 … ON, O is the sequence of observable symbols.
- Pi: pi represents the initial state probability. The initial state probability normally is used to identify the probability of starting at a particular hidden state H1.
- T: ti,j, T is the hidden state transition probabilities where ti,j = P(tk = Hj—tk-1 = Hi) represents the probability of transitioning from hidden state Hi to hidden state Hj.
- E: ei(k) where ei(k) = P(Ok—tk = Hi) represents the probability of an observable symbol k emitted from state Hi.

Overall, an HMM can be represented using notation L = A, B, Pi.

## Enhanced handover mechanism using mobility prediction (eHMP)

The proposed eHMP enhances the selection of WiFi AP and optimization on the usage of multiple network interfaces that are available for mobile devices in the handover process.

**Fig 10:**
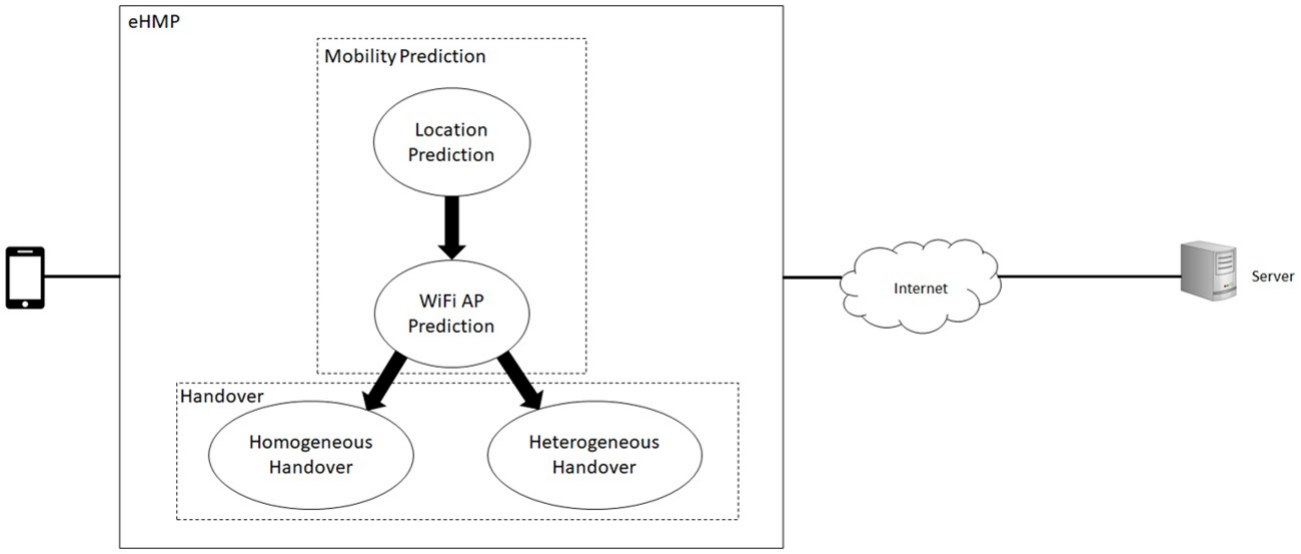
Enhanced handover mechanism using mobility prediction.

eHMP is composed of two phases, namely, mobility prediction and handover. The mechanism first considers the predicted geographical location of the mobile user by referring to the historical path that had been visited by the mobile user. The availability of the mobile user’s location enables the mobile device to intelligently connect to the optimal network either through WiFi or the cellular network or the handover from WiFi to the cellular network or vice versa. The output from the prediction will be used for heterogeneous handover from WiFi to the cellular network or vice versa. Fig 10 shows an overview of proposed eHMP.

**Fig 11:**
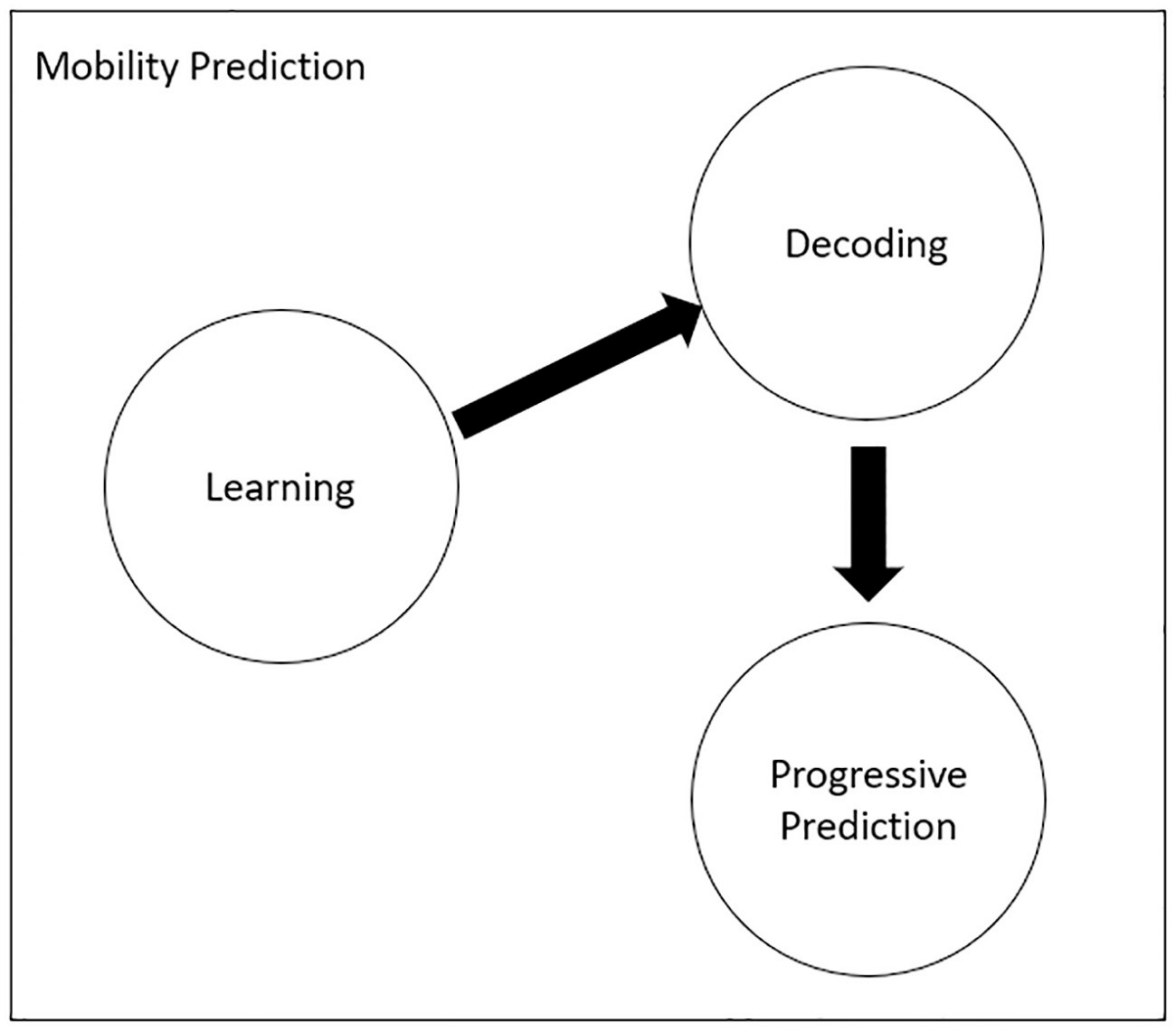
Mobility prediction stages.

Unlike existing mobility prediction algorithms that only consist of learning and decoding stages, eHMP deploys additional progressive mobility prediction, as shown in Fig 11. The output from the learning stage is decoded and utilized by the progressive prediction so that the mobile device can learn the user’s behaviour and optimize the network selection process.

There are two hidden states and two observable states from the adoption of the dual HMM in mobility prediction. The parameters that link the hidden states and observable states are known as transmission and emission probability. Transmission probability shows the transition from one hidden state to another hidden state, whereas emission probability is the emission of the observable state from the hidden state. To maximize the probability of occurrence of the observation sequence and joint probability of observation sequence and hidden state sequence, training of the HMM parameters is required in the learning stage.

**Fig 12:**
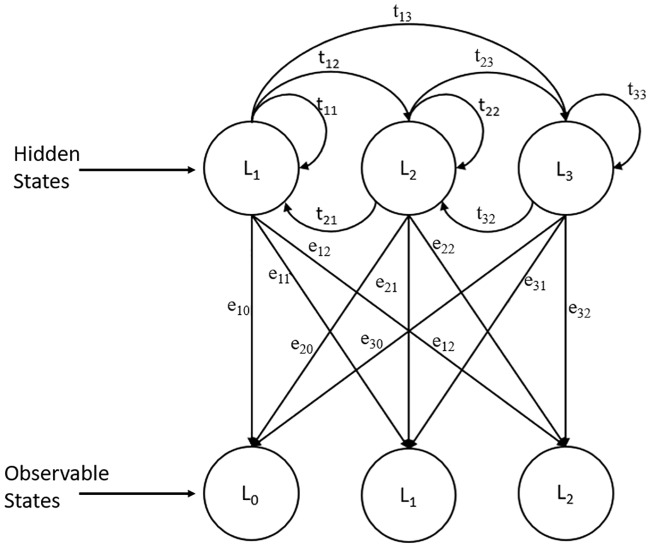
First HMM represented by location.

In the first HMM, as illustrated in Fig 12, the hidden state represents the future geographical coordinate of mobile users, while the observable state is the current geographical coordinate of mobile users. Emission probabilities *e*_21_ describe the probability that the mobile user was at location 1 at time n-1 when the mobile user is at location 2 at time n. On the other hand, the transition probabilities *t*_23_ describe the probability of the mobile user moving from location 2 at time n to location 3 at time n+1.

By using the historical location of the mobile user in which the mobile user’s geographical coordinate location is recorded in a pair (for example, (location 2, location 1), where the user is walking from location 1 to location 2) and emission probabilities P(e) are computed by using Eq 1, while the transition probability is computed using Eq 2.

$$\begin{array}{c} P\left(e\right)=\frac{L_{2}|L_{1}}{N(L_{2})} \end{array}$$ (1)

where *P*(*e*) is the emission probability, *L*_2_|*L*_1_ is the number of visits of location 2 after visiting location 1, and *N*(*L*_2_) is the number of occurrences of location 2.

$$\begin{array}{c} P\left(t\right)=\frac{L_{3}|L_{2}}{M(L_{2})} \end{array}$$ (2)

where *P*(*t*) is the transition probability, *L*_3_|*L*_2_ is the number of occurrences of moving to location 3 from location 2, and *M*(*L*_2_) is the number of movements from location 2.

**Fig 13:**
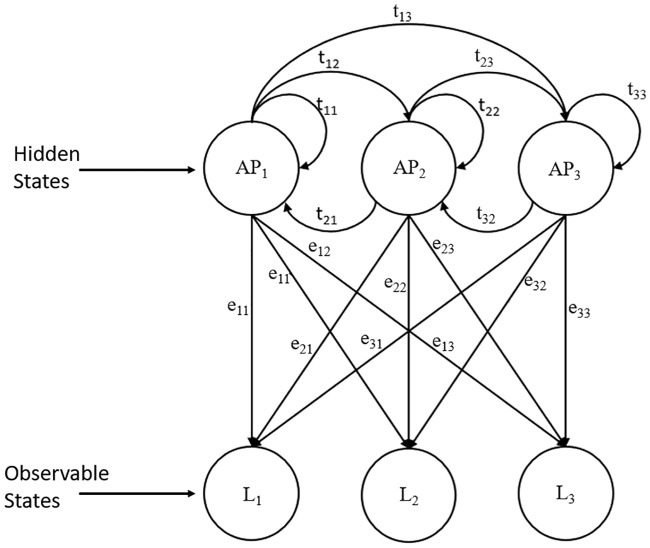
Second HMM represented by location and WiFi AP.

Fig 13 illustrates the second HMM used. The second HMM is used to predict the optimal WiFi AP based on the mobile user location. The hidden state in the second HMM represents the optimal WiFi AP that is nearest to the observable state, in which the observable state represents the geographical location of the mobile user. Emission probabilities *e*_11_ in the second HMM describe the probability that a mobile user is at location 1 when the optimal WiFi AP 1 should be connected, while transition probabilities *t*_12_ describe the probability of handover from the optimal WiFi AP 1 to the optimal WiFi AP 2.

Differing from the first HMM, the learning phase of second HMM parameters require distance computation in order to obtain the optimal WiFi AP that should be connected by the mobile user at each geographical location. The optimal WiFi AP is required to be computed because the conventional WiFi in mobile devices often encounters issues such as disconnection before connecting to the neighboring WiFi AP. This situation causes mobile devices to consume more power during the frequent handover process. Mobile devices constantly increase the signal power in order to maintain the WiFi connection with the deteriorating WiFi AP and rescan for available APs before total disconnection with the previously connected WiFi AP [21]. This process indirectly causes energy wastage and lowers mobile users’ quality of experience (QoE). Thus, the proposed mobility prediction helps to preserve resources and provide better network connectivity.

The distance between the WiFi AP and a mobile user is computed using Eqs 3, 4 and 5. Using the equations, the WiFi AP that has the lowest distance to each geographical coordinate of the mobile user will be recorded in a pair, for example, (WiFi AP1, location 1). Then, transition and emission probabilities can be calculated.

$$\begin{array}{c} a={\text{sin}}^{2}\left(\Delta \phi /2\right)+\text{cos}\;\phi_{1}\cdot \text{cos}\;\phi_{2}\cdot {\text{sin}}^{2}\left(\Delta \lambda /2\right) \end{array}$$ (3)

$$\begin{array}{c} c=2\cdot atan2(\sqrt{a},\sqrt{\left(1-a\right)}) \end{array}$$ (4)

$$\begin{array}{c} d=R\cdot c \end{array}$$ (5)

where a is the square of half the chord length between two points, c is the angular distance in radians, d is distance, *ϕ* is latitude, λ is longitude, and R is the Earth’s radius (mean radius = 6.371 km).

After computing the list of the optimal WiFi APs based on the mobile user geographical coordinates, the emission probability and transmission probability are computed using 6 and 7, respectively.

$$\begin{array}{c} P\left(e\right)=\frac{AP_{1}|L_{1}}{N(AP_{1})} \end{array}$$ (6)

where *P*(*e*) is the emission probability, *AP*_1_—*L*_1_ shows the number of connections to the optimal WiFi *AP*_1_ if the mobile user is at location *L*_1_, and *N*(*AP*_1_) is the total number of connections to *AP*_1_.

$$\begin{array}{c} P\left(t\right)=\frac{AP_{2}|AP_{1}}{H(AP_{1})} \end{array}$$ (7)

where *P*(*t*) is the transition probability, *AP*_*b*_—*AP*_*a*_ is the number of occurrences of handovers from *AP*_1_ to *AP*_2_, and *H*(*AP*_1_) is the number of handovers from *AP*_2_.

**Fig 14:**
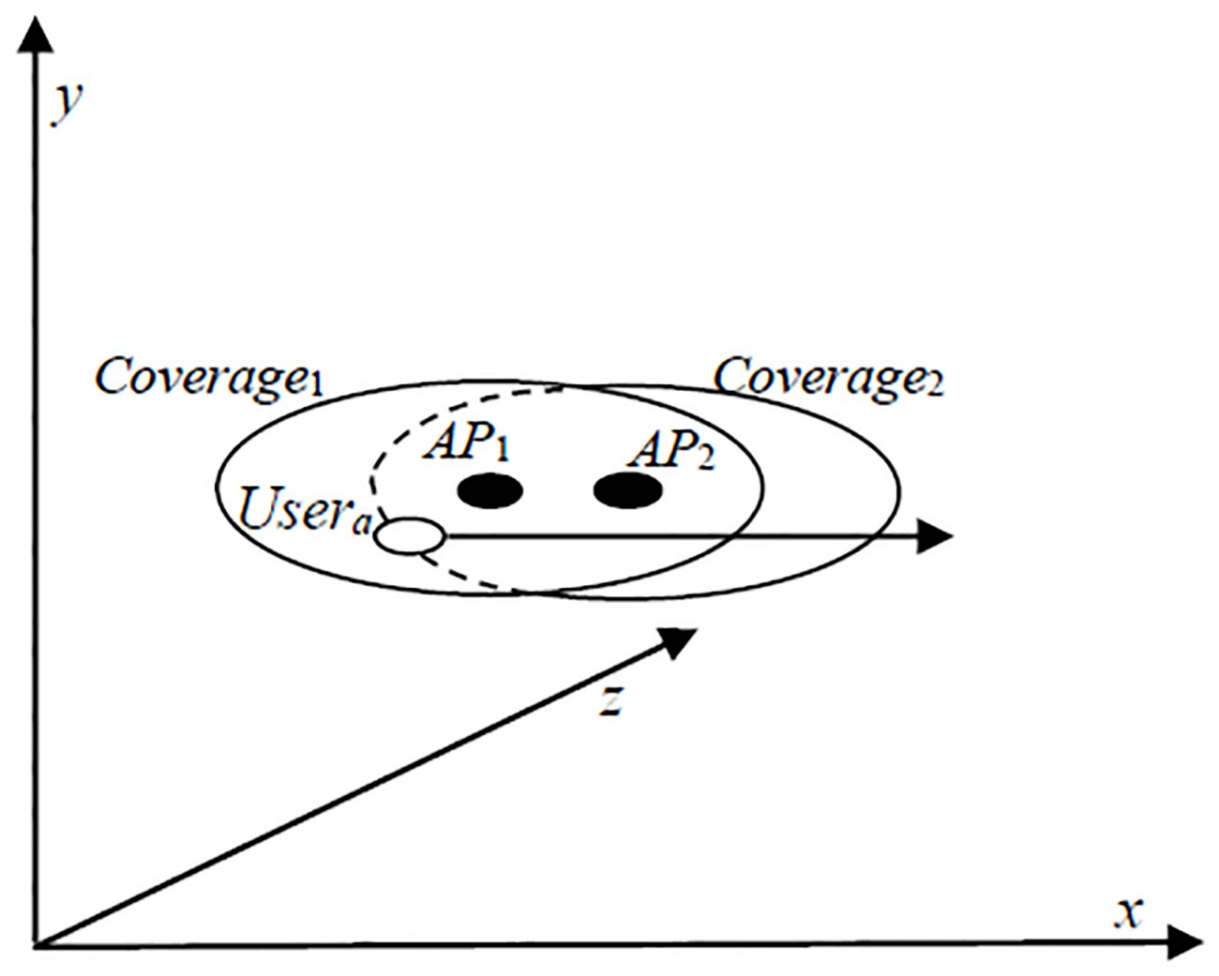
Visualization of WiFi signal strength.

Fig 14 shows an example of WiFi connectivity in the mobile environment. As depicted, two WiFi APs are available to be connected, and the user is initially connected to AP1. If mobile users constantly move from *AP*_1_ to *AP*_2_, users face problems, such as the problem encountered when the device is always in the high energy state due to repeated scan and association requests with a low signal WiFi AP before losing the connectivity. Upon disconnecting from *AP*_1_, the user will be connected to *AP*_2_. However, due to constant movement of the user, the mobile device tends to be in the high energy state when there are repeated scans for availability and association requests as it is leaving the signal coverage of *AP*_2_.

Although two WiFi APs are located very close to each other, the WiFi AP supports the multiple channel capability that helps to reduce interference and improve the WiFi signal. As such, the mobile device will still enter the high energy state due to connection to the non-optimal WiFi AP.

The proposed eHMP makes the prediction based on the observable state sequence and parameters such as transition and emission probabilities, which is learned from the previous learning stage. Since the dual HMM is used in the proposed mechanism, there are two predictions made from each HMM.

In the first HMM, the future geographical location of mobile users is predicted, which is represented by the hidden state. Nevertheless, the prediction is made based on information such as the mobile user geographical location represented by the observable state and transmission and emission probabilities that are learned from the mobile user history location. With this information, mobile devices decode the hidden state, which is the mobile user’s geographical coordinate with the Viterbi algorithm. The Viterbi algorithm is used to compute the most probable extensions of paths that lead to the current cell. For a given state q(j) at time t, the eHMP mechanism will compute the value v(j) as depicted in 8.

$$\begin{array}{c} v_{t}\left(j\right)=\overset{N}{\underset{i=1}{\text{max}}}\;v_{t-1}\left(i\right)a_{ij}b_{j}\left(o_{t}\right) \end{array}$$ (8)

The three factors, which are multiplied in Eq 8 to compute the Viterbi probability in time *t*, are

- *v*_*t*−1_(i) represents the Viterbi probability from the previous time step
- *a*_*ij*_ represents the transition probability from the previous state *q*_*i*_ to the current state *q*_*j*_
- *b*_*j*_(*o*_*t*_) represents the emission probability that state j emits observable *o*_*t*_

To obtain the best path according to the observable state sequence, Eq 9 is used when *t* = 1 and Eq 10 is used when *t* = 2 to compute the Viterbi probability; moreover, Eq 11 is the best state sequence, which keeps track of the path of hidden states that leads to each observable state.

$$\begin{array}{c} v_{t}\left(j\right)=a_{0j}b_{j}\left(o_{1}\right);1\leq j\leq N \end{array}$$ (9)

$$\begin{array}{c} v_{t}\left(j\right)=\overset{N}{\underset{i=1}{\text{max}}}\;v_{t-1}\left(i\right)a_{ij}b_{j}\left(o_{t}\right);1\leq j\leq N,1<t\leq T \end{array}$$ (10)

$$\begin{array}{c} bt_{t}\left(j\right)=\text{arg}\;\overset{N}{\underset{i=1}{\text{max}}}\;v_{t-1}\left(i\right)a_{ij}b_{j}\left(o_{t}\right);1\leq j\leq N,1<t\leq T \end{array}$$ (11)

Since the sequence of the mobile user’s geographical location is required in order to decode the sequence of hidden states, which depicts the future geographical location using the Viterbi algorithm, this algorithm depends on the input of the observable state sequence to generate the required output. However, this approach is insufficient, as mobility prediction should always be able to forecast a few seconds ahead. For example, to predict where the mobile user is at t = n, the Viterbi algorithm requires the mobile user location t = n-1.

The existing HMM decoding problem relies on the availability of the observation sequence in order to decode the hidden state sequence. In reality, the mobile user’s geographical coordinate will only be available if the mobile user is currently at that location. Thus, the progressive mobility prediction (PMP) is used, where prediction can be made with only one mobile user geographical coordinate. The proposed PMP predicts up to five time steps into the future, where each time step is equivalent to five seconds.

**Fig 15:**
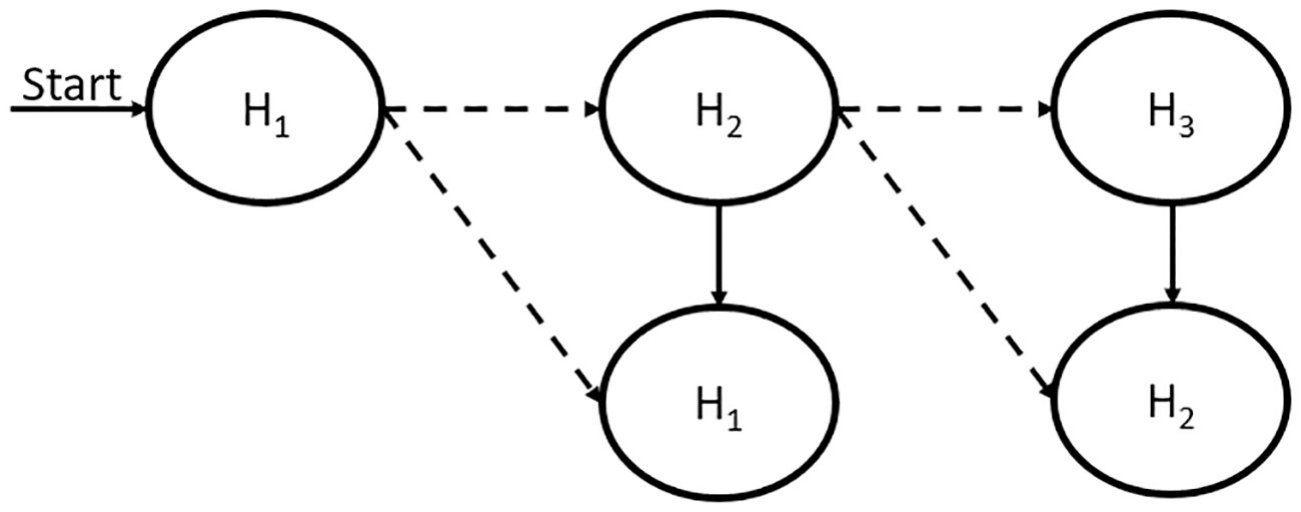
Proposed progressive mobility prediction.

To perform the first forecast in the first HMM, the mobile user’s first geographical coordinate will be used as the first hidden state in the model. This is followed by placing the first hidden state as the next observable state input to the model. By using this approach, PMP can be made by feeding the input into the model whenever the hidden state can be decoded using the available observable state. Fig 15 shows the utilization of the first hidden state decoded as the next observable state to obtain the second hidden state.

**Fig 16:**
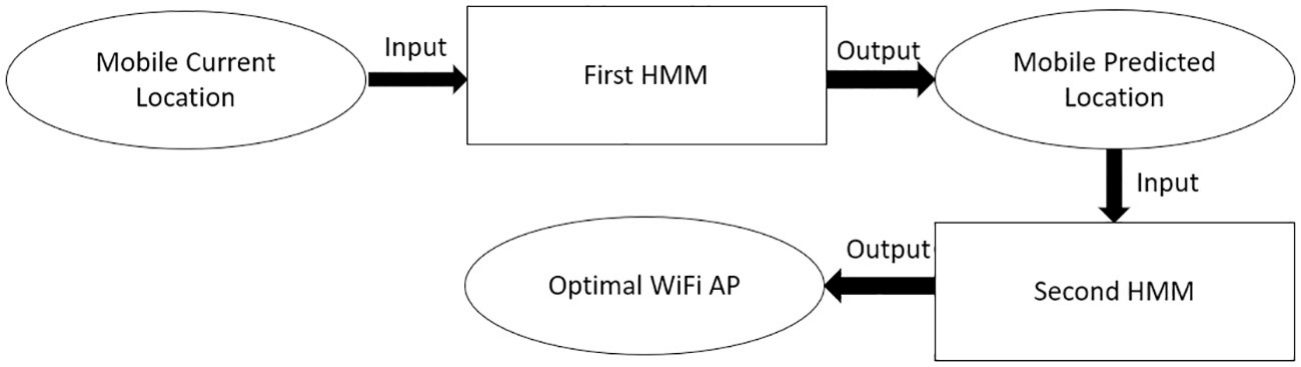
Example of the dual hidden Markov model.

The prediction made in the second HMM is the optimal WiFi AP in future timestamps based on the available observation state such as the geographical coordinate of the user. This prediction enables users to connect to the best signal level WiFi AP at a geographical location, thus reducing the power consumption device as well as increasing QoE. Prediction should be always forecast a few seconds ahead, such that the future location of the mobile user should be predicted and hence can be utilized as the input to the second HMM in order to predict the optimal WiFi AP to be connected at that predicted location. Fig 16 shows the utilization of the first HMM output as the input for the second HMM prediction.

After the mobility prediction phase performs progressive prediction, in which the location and optimal WiFi AP are predicted, handover will be performed by the mobile device. If the mobile device only has a WiFi interface for data communication with the server, handover is performed based on the output from mobility prediction, whereas if the predicted WiFi AP is different from the currently connected WiFi AP, a homogeneous handover between the WiFi APs will be performed. Meanwhile, if the mobile device uses MPTCP that supports the usage of the multipath between the mobile device and server, a heterogeneous handover will be performed between the WiFi interface and cellular interface.

### Homogeneous handover

In this research, the homogeneous handover will be performed between the WiFi APs. The output of PMP shows the optimal WiFi AP that should be connected by the mobile user based on the user’s geographical coordinate. Hence, if the currently connected WiFi AP is different from the output of PMP, the handover between WiFi APs is needed.

**Fig 17:**
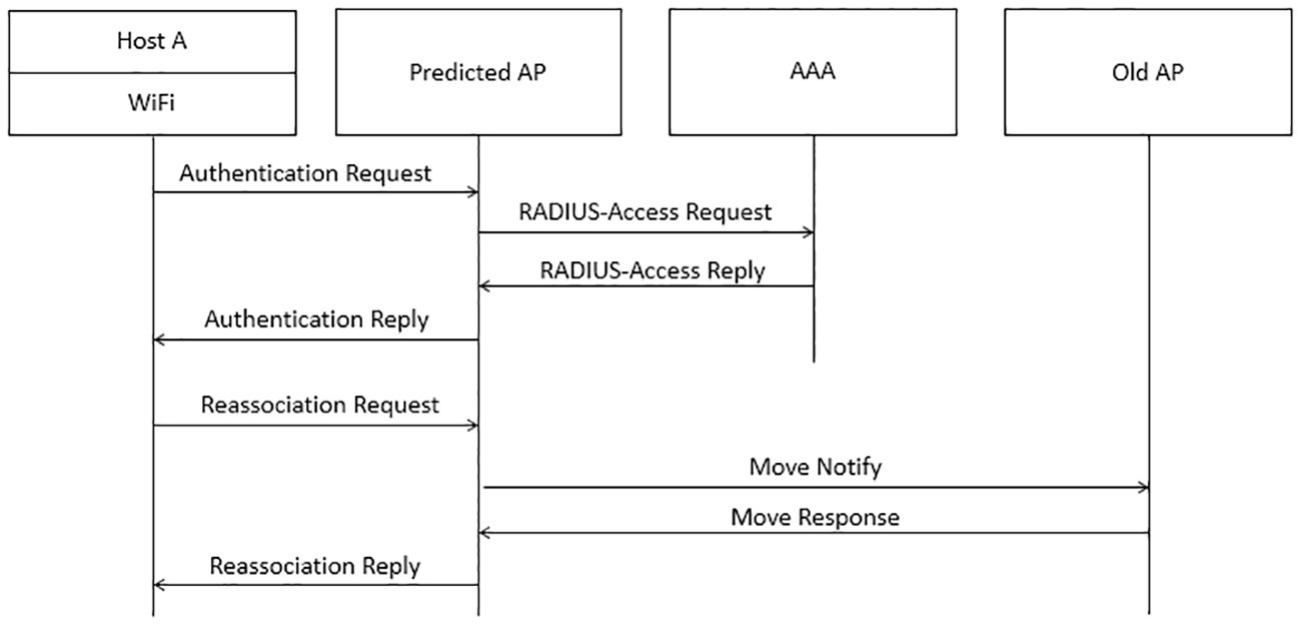
Homogeneous handover between WiFi APs.

Fig 17 shows that host A is performing homogeneous handover between WiFi APs after receiving the output of PMP which has a different WiFi AP compared to the currently connected WiFi AP. There are three steps in the homogeneous handover. The first step requires Host A to disconnect from the currently connected WiFi AP. Next, Host A sends an authentication request to the predicted WiFi AP in order to authenticate with the authentication, authorization and accounting (AAA) server. After Host A is successfully authenticated, the reassociation request frame that includes the old AP information will be sent to the newly connected WiFi AP. The newly connected WiFi AP will communicate with the old AP to verify host A was previously associated. If host A was previously associated, any buffered frames at the old AP will be transferred to the new AP.

### Heterogeneous handover

The heterogeneous handover describes the change in different underlying network communication accesses, such as from WiFi to cellular or from cellular to WiFi. Fig 16 illustrates the output from the second HMM, which is the AP prediction showing the optimal WiFi AP that should be connected by a mobile user at each predicted location. According to this output, the mobile device can actually decide whether a heterogeneous handover from WiFi to cellular is required if multiple networks are available in the mobile device.

For example, if the output of the second HMM shows there is a difference between the currently connected AP and the predicted AP, the mobile device will initiate a heterogeneous handover from WiFi to the cellular network in order to prevent network service disruption. Only after the predicted AP is connected by mobile device will a heterogeneous handover from the cellular network to WiFi be initiated in order to offload the service.

The current mobile network architecture does not support seamless handover because changes in the connected network will cause service disruption. For example, downloading a large-size file will encounter failure if there is a change in the network. This is due to the underlying communication protocol that is used, such as TCP, where each end is bound with one IP address. If there is a change in the network, the IP in the mobile device will change as well, which leads to the download failure, and the user will have to restart the download again.

To perform seamless heterogeneous handover without causing network disruption, MPTCP is implemented in the handover process and supports the change in path priority to either regular or backup. A regular path is used for normal data flow, while a backup path will only be used when no regular paths are available. The mobile device should only use one network interface at a time in order to prevent the wastage of data quota, especially cellular quota. Thus, the mobile cellular network interface will always act as a backup path to all outgoing connections, while the WiFi network interface will be a regular path for all network data transfers.

**Fig 18:**
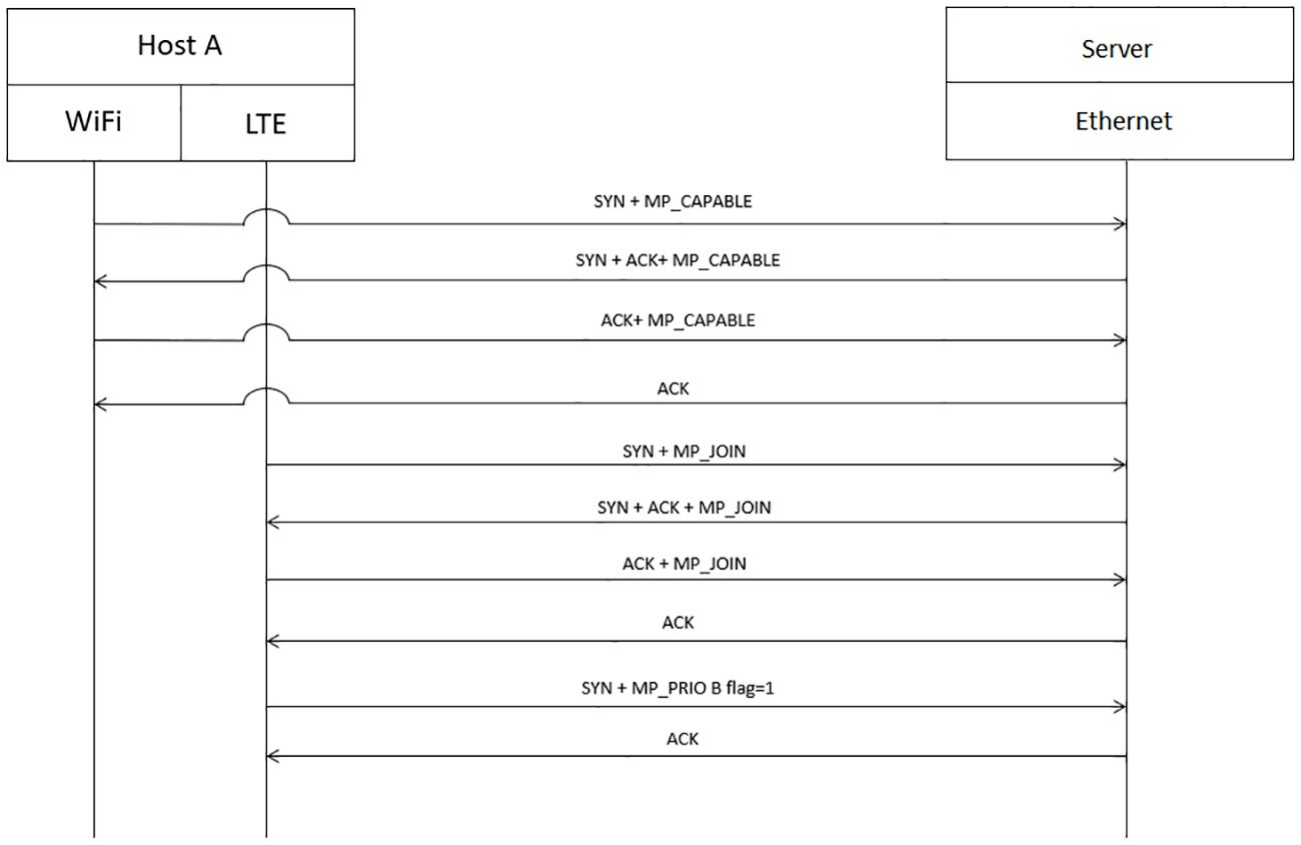
Seamless heterogeneous handover.

Fig 18 shows an example of the sequence diagram of two communicating hosts A and servers. The connection initiated using the “MP CAPABLE” option in the packet to inquire whether the server is supporting MPTCP. The server reply acknowledges by sending “MP CAPABLE” to indicate that MPTCP is supported. Then, the available path such as the cellular network, LTE, can send the request to the server to join the connection for aggregating the connection. As depicted in Fig 18, the LTE path will be used as the backup path by sending “MP PRIO” with the B flag value of 1 to the server. After “MP PRIO” is received by the server, LTE will only be used from both ends if there are no paths available.

**Fig 19:**
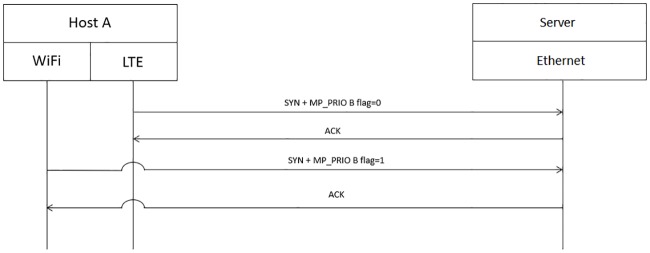
Use of the cellular network as the primary network path.

**Fig 20:**
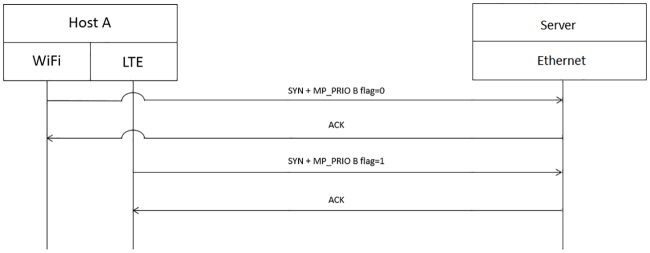
Use of the WiFi network as the primary network path.

PMP predicts the optimal WiFi AP that should be connected by a mobile user. If the output of PMP is different from the currently connected WiFi AP, the cellular path will be used as the primary path by using the cellular interface to send “MP PRIO” with the B flag value of 0 to server for indicating the usage of the path, as depicted in Fig 19. Only when the output of PMP is the same as the currently connected WiFi AP will host A send “MP PRIO” with the B flag value of 1 from the cellular interface to the server to stop using the cellular network. Then, WiFi will now be used as the primary path, as depicted in Fig 20.

## Results

### Homogeneous network performance

**Fig 21:**
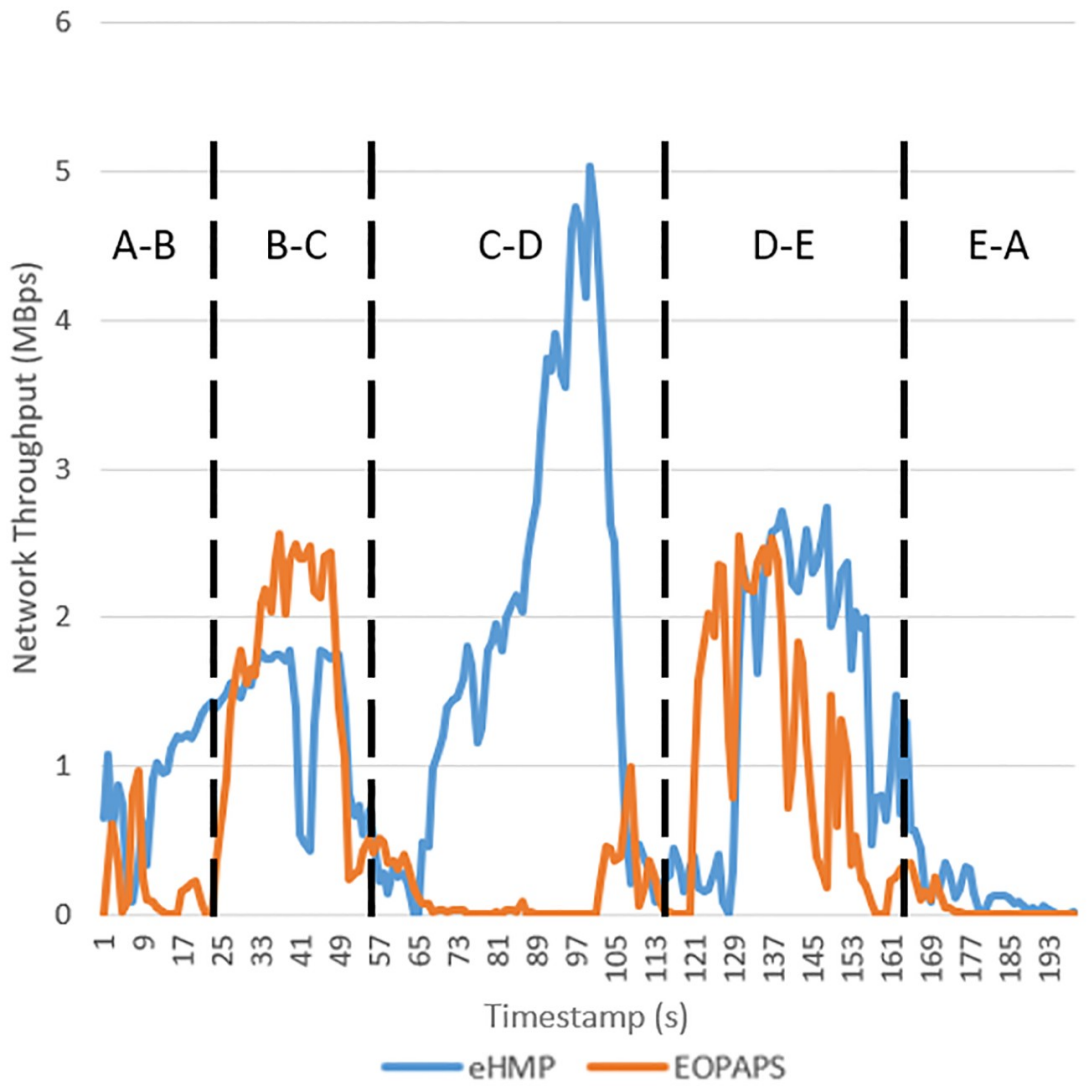
Comparison of network throughput between eHMP and EOPAPS.

Fig 21 illustrates the network throughput of EOPAPS and eHMP in the homogeneous architecture, and the average network throughput of eHMP and EOPAPS is displayed in Table 1. Between points A and B, the average throughput for EOPAPS is 0.22 MBps, lower than that for eHMP, which achieves an average of 0.94 MBps even though the RSSI is higher. This result is because the experiment was not done concurrently; thus, the bandwidth provided by the WiFi AP is different. During the testing of EOPAPS, the WiFi AP is congested, causing degradation in the network throughput. From points B-C, the average throughput for the existing EOPAPS and eHMP is comparable, which is 1.71 MBps and 1.36 MBps, respectively. Although eHMP does not increase the average throughput, it does not degrade the performance when transferring the file. The difference in terms of the average throughput is due to the different bandwidths provided by the AP when conducting the experiment. From points C-D, eHMP showed a major improvement by providing 1.8 MBps average throughput compared to that of the existing EOPAPS, with an average throughput of 0.15 MBps. The increase in the average throughput by 1098% is because the mobile device that is using EOPAPS is still connected to WiFi 2 even when the RSSI is low, whereas eHMP predicted that the mobile device is moving towards WiFi 3 coverage, hence triggering the handover process to WiFi 3 before the signal degrades further. From points D-E, the improvement in terms of average throughput observed is 35%. eHMP predicted that the mobile device will be moving towards WiFi 4, thus triggering the handover process to the new WiFi AP before the signal degrades further. EOPAPS only triggers the handover process for the mobile device to connect to WiFi 4 in D-E when the RSSI is too low, which causes a lower average throughput when EOPAPS is used. From E-A, the average throughput is low for both eHMP and EOPAPS. The reason is that there is a blind spot for the WiFi AP, where EOPAPS yields an average of 0.04 MBps throughput, while eHMP yields 0.14 MBps. The mobile device is unable to continue downloading the file because it is unable to connect to any WiFi AP. Nevertheless, the download process continues earlier when eHMP is used because the handover process is triggered earlier due to the prediction model that is implemented in eHMP. This result shows that eHMP can assist the handover process even when the network design is not optimal.

**Table 1:** Average throughput between eHMP and EOPAPS in each range.

Range	eHMP (MBps)	EOPAPS (MBps)
A-B	0.9372	0.2186
B-C	1.3588	1.7122
C-D	1.8031	0.1505
D-E	1.4394	1.0671
E-A	0.1369	0.0360

**Fig 22:**
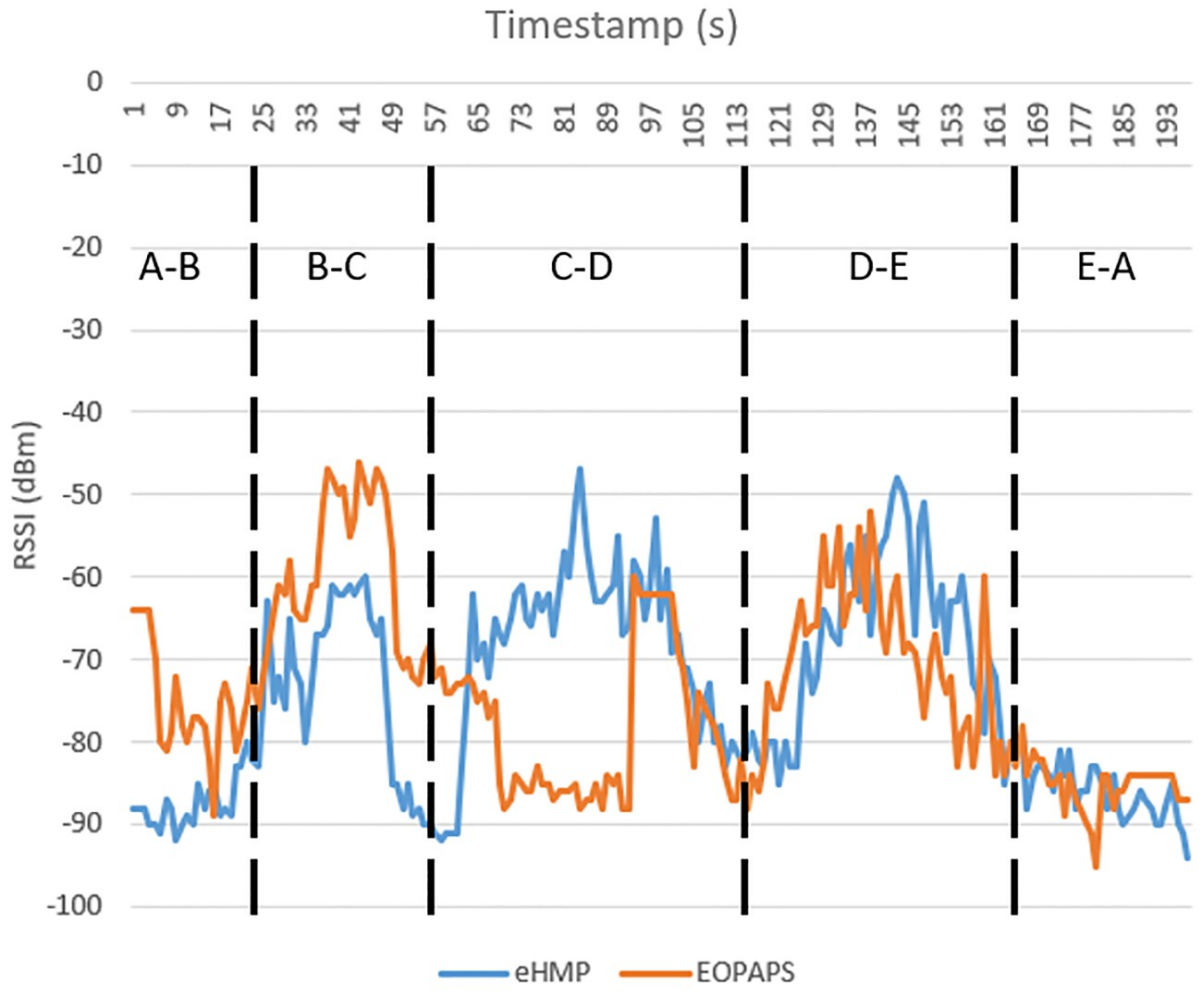
Instantaneous RSSI of EOPAPS and the proposed eHMP.

Fig 22 shows the RSSI of the mobile device during testing of the testbed. Between A-B and B-C, the RSSI of EOPAPS is higher than that of eHMP. This situation is caused by temporary interference between the mobile device and the WiFi AP, as the two experiments are performed at different times. Even though eHMP has a lower RSSI than that of EOPAPS, the network throughput was not affected tremendously. From C-D and E-D, eHMP has a higher RSSI than that of EOPAPS, which is because eHMP triggers the handover process to the optimal WiFi AP before the RSSI degrades further. The optimal WiFi AP was obtained from the prediction mechanism implemented in eHMP. EOPAPS only triggers the handover process when EOPAPS is disconnected from WiFi 3. Between points E and A, both EOPAPS and eHMP showed a low RSSI. This happened because of the blind spot between the WiFi APs, causing the mobile device’s inability to connect to any WiFi AP. As such, the downloading process stopped at this stage. Nevertheless, the results showed that eHMP assisted the handover process even when the WiFi network design was not optimal.

**Fig 23:**
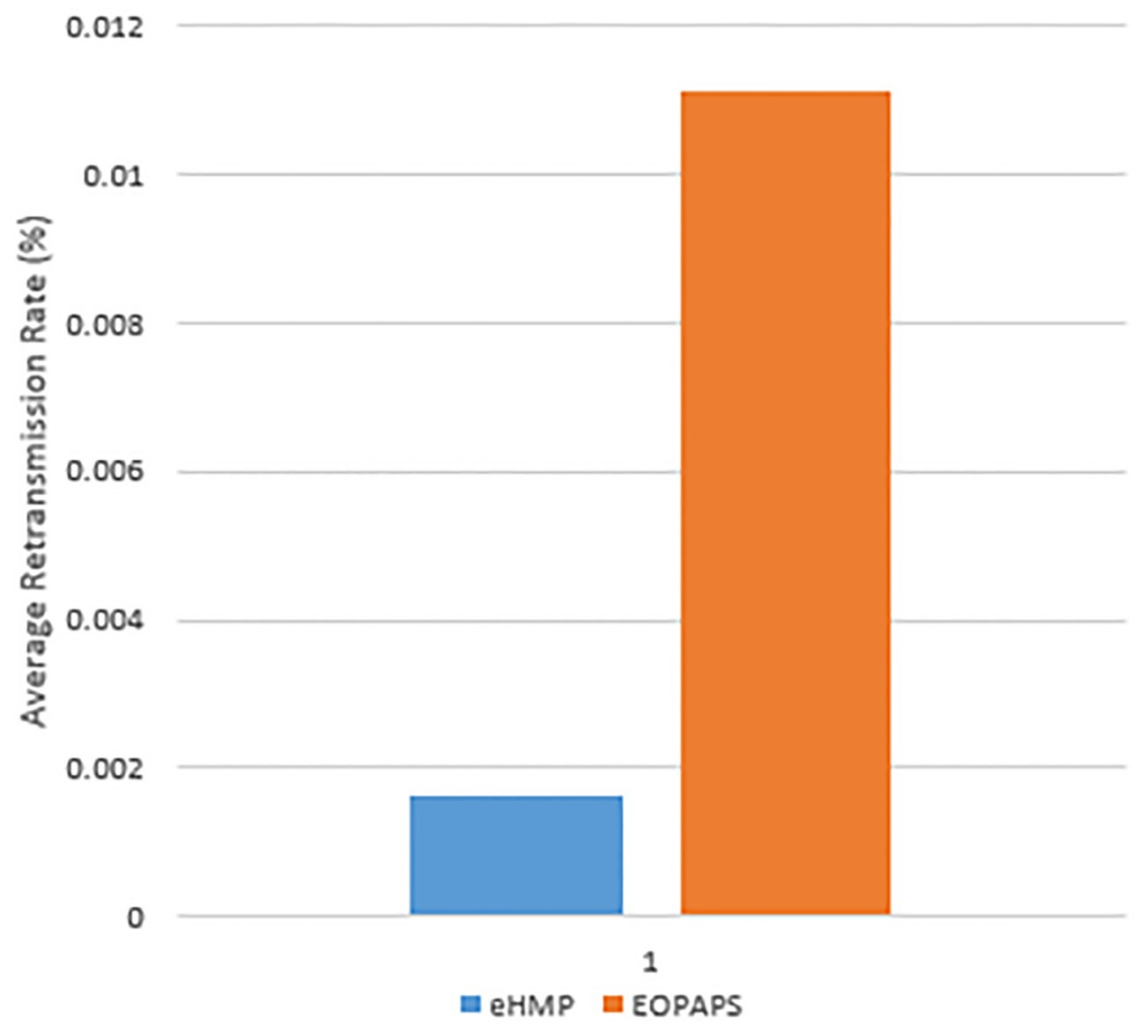
The retransmission rate using eHMP and EOPAPS.

Fig 23 shows the retransmission rate for EOPAPS and eHMP. The retransmission rate decreases by 85% when eHMP is used. The retransmission rate is lower than that for EOPAPS because eHMP manages to trigger the handover process in order to maintain the connectivity. Thus, retransmission from the server decreases, whereby the server is able to receive acknowledgement from the mobile device and continue to send the next packet. When the mobile device handover occurs in the random surrounding WiFi AP, poor network connectivity causes the server’s inability to receive the acknowledgement message from the mobile device. As such, the server retransmits the TCP packets until the mobile device successfully sends the acknowledgement message to the server.

### Heterogeneous network performance

To compare the performance of the heterogeneous handover with different approaches, two different experiments were conducted, namely, heterogeneous handover using MPTCP and heterogeneous handover using eHMP. Performance metrics such as the network throughput RSSI and retransmission rate are compared.

**Fig 24:**
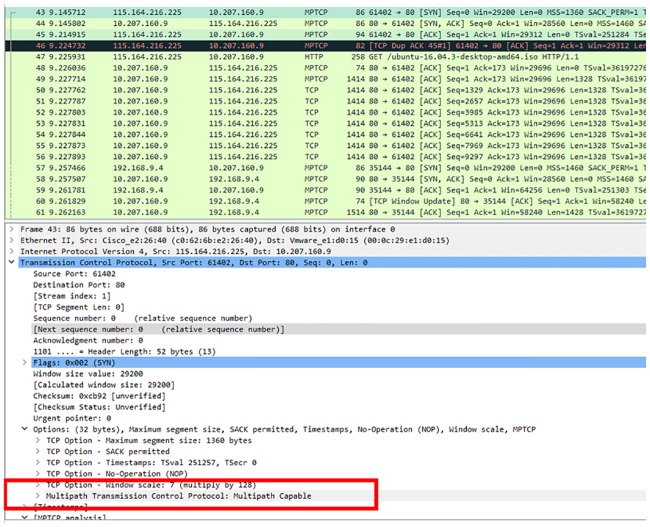
MPTCP capable message exchange between the mobile device and server.

For the heterogeneous handover to be used, MPTCP was implemented in the mechanism so that when handover is triggered by mobile device, the same TCP session will be used instead of starting a new TCP session after handover succeeds. Fig 24 shows the multipath-capable TCP option message exchange between the mobile device and server. The mobile device sends the multipath-capable message to the server to check if the server is supporting MPTCP so that the mobile device can use both cellular and WiFi interfaces to communicate with server. For both mobile devices and servers to use MPTCP, the server replies ACK to the mobile device to announce that it supports MPTCP.

**Fig 25:**
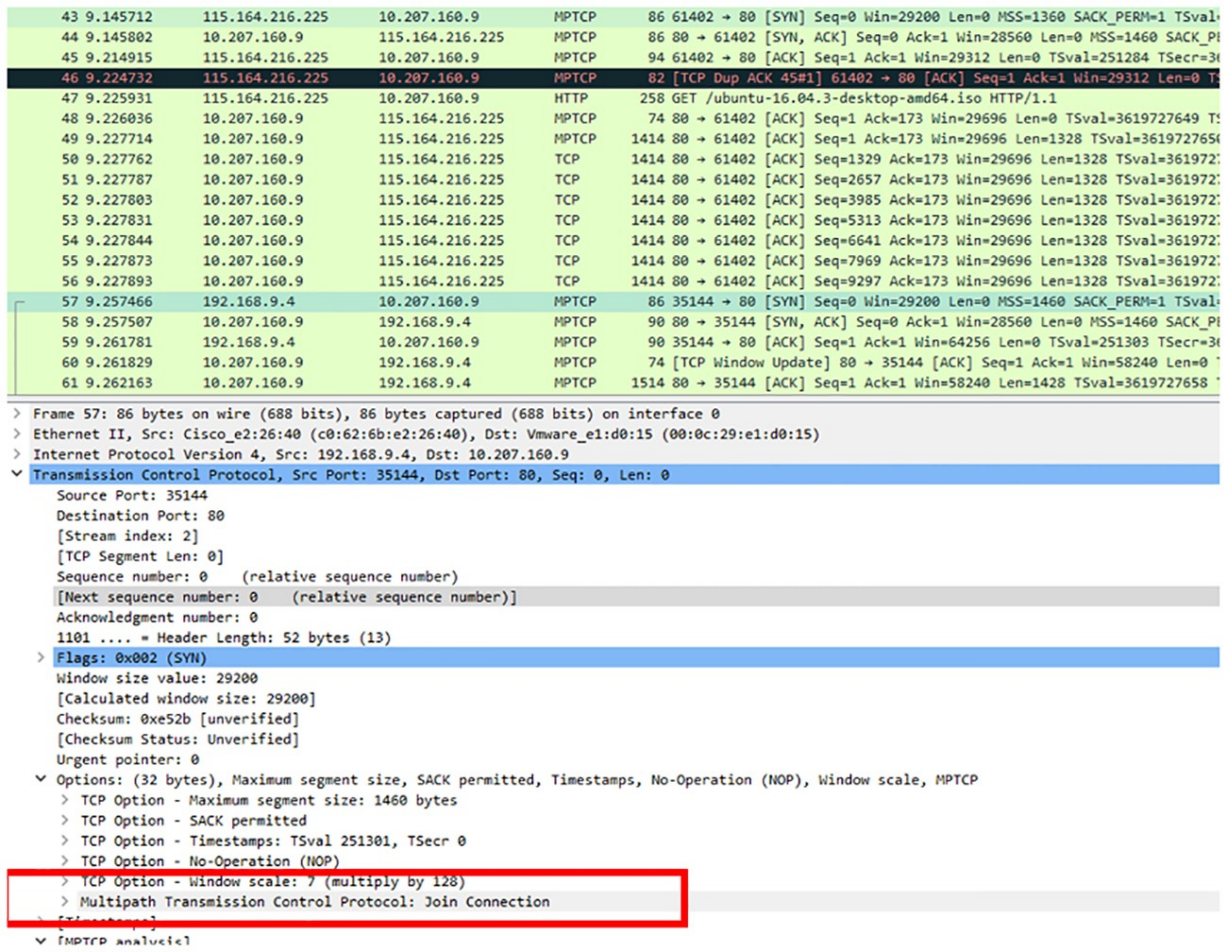
New subflow for data communication.

Fig 25 shows the join connection MPTCP option. The message shows that MPTCP was enabled and that the mobile device can create a new subflow with the server. After the mobile device successfully communicates with the server through the cellular interface, the mobile device sends the join connection through the WiFi interface to the server in order to add WiFi as the new subflow to communicate with the server. The server replies ACK to the mobile device WiFi interface to acknowledge the subflow connection so that the mobile device can communicate with the server through the cellular and WiFi interfaces.

**Fig 26:**
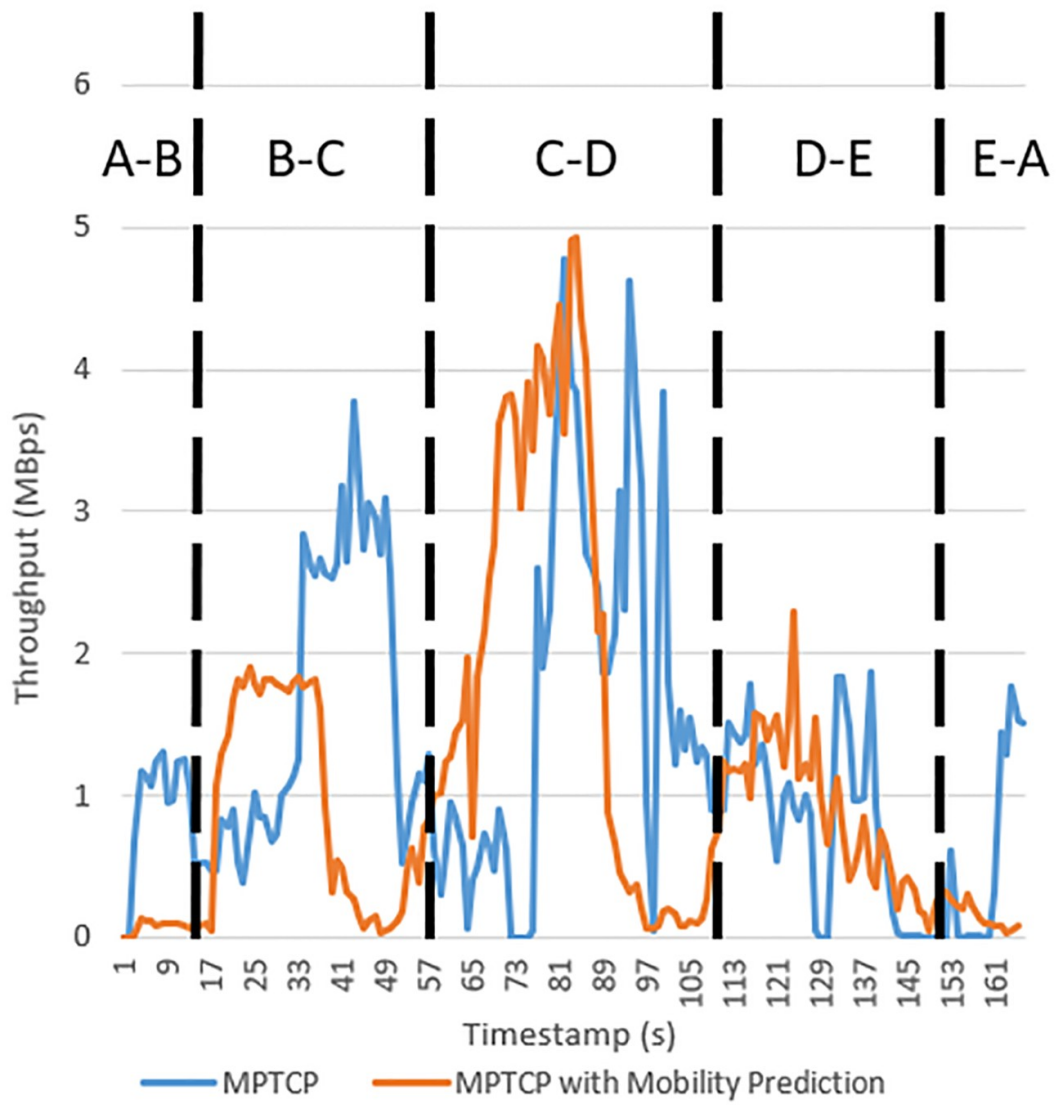
The network throughput of MPTCP and MPTCP with mobility prediction.

Fig 26 shows the throughput when using MPTCP and MPTCP with mobility prediction. As shown in Fig 26, MPTCP with mobility prediction shows low throughput between points A and B. This result may occur because the kernel is still using cellular as the primary network path instead of using WiFi for downloading, and the cellular network has low network throughput at that time. Between points B and C, the proposed MPTCP with mobility prediction successfully triggers the handover process to connect to WiFi 2, which can provide better network performance for the mobile device. When the mobility prediction model is used together with MPTCP, the network throughput does not improve, which is due to the poor network throughput observed in the cellular network path. As shown in Table 2, the average throughput observed in MPTCP from points B-C is 0.87 MBps, whereas MPTCP with mobility prediction achieves only 0.06 MBps. This result occurs because the experiment was not done concurrently; thus, the bandwidth provided by the WiFi AP and cellular is different. Meanwhile, compared to MPTCP, MPTCP with mobility prediction yields higher throughput between C and D, whereby the average throughput increased by 13%. This is because the proposed mobility prediction predicted the optimal WiFi AP for the mobile device; hence, the mobile device performs handover to the predicted WiFi AP in a faster manner than that of MPTCP. Similarly, higher network throughput is observed when MPTCP with mobility prediction is used between points D and E, where the average throughput is increased by 11%. This is due to the timely WiFi AP handover when eHMP predicted that the mobile user will enter the network coverage of WiFi 4 from D-E. As shown in Fig 26, the mobile device is able to connect to WiFi 4 when MPTCP with mobility prediction is used, causing the increase in network throughput. Both MPTCP and MPTCP with mobility prediction have low throughput from E-A because the mobile device is unable to connect to the internet due to the blind spot of WiFi AP.

**Table 2:** Average throughput of MPTCP and MPTCP with mobility prediction in each range.

Range	MPTCP (MBps)	MPTCP with Mobility Prediction
A-B	0.8760	0.0826
B-C	1.6568	0.9807
C-D	1.6859	1.9016
D-E	0.7973	0.8871
E-A	0.5719	0.1612

**Fig 27:**
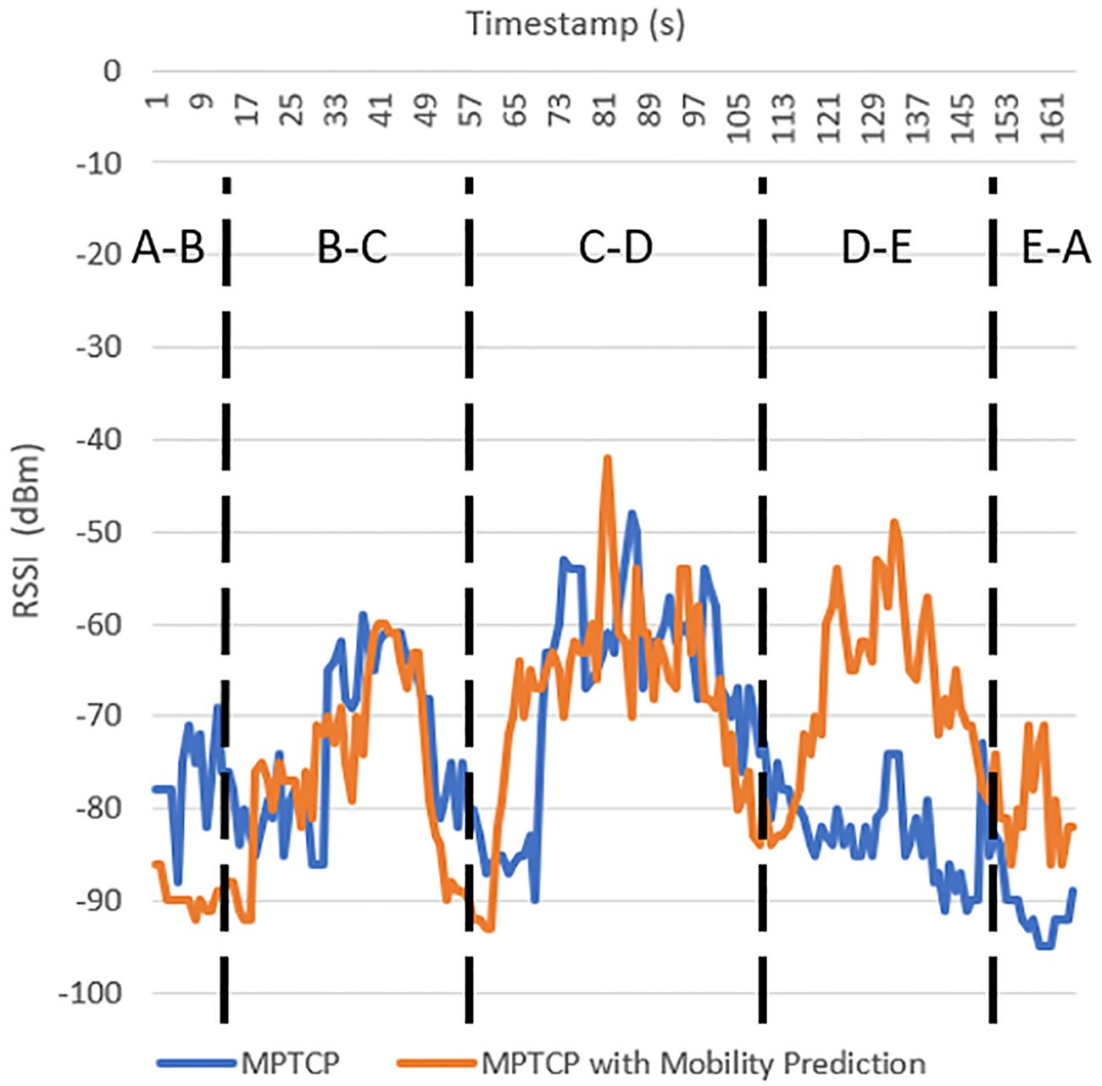
RSSI of connected WiFi AP in MPTCP and MPTCP with mobility prediction.

Fig 27 shows the RSSI of the connected WiFi AP in MPTCP and MPTCP with mobility prediction. Between points A and B, the RSSI is lower when MPTCP with mobility prediction is used, which is because of network congestion in the WiFi AP in this situation. MPTCP with mobility prediction shows higher RSSI during the beginning of B-C, which is because the mobility prediction model assisted the mobile device to handover to the predicted WiFi AP compared to the existing MPTCP that still connected to the previous WiFi AP. Similarly, C-D shows that the proposed MPTCP with mobility prediction yields a higher RSSI at the beginning. Although MPTCP can increase the network performance, the intelligence that is added through mobility prediction can assist the mobile device in achieving a better QoS. From D-E and E-A, the existing MPTCP fails to handover to the optimal WiFi AP, which leads to a low RSSI compared to that with the proposed MPTCP with mobility prediction, in which the mobile device perceives a high RSSI.

**Fig 28:**
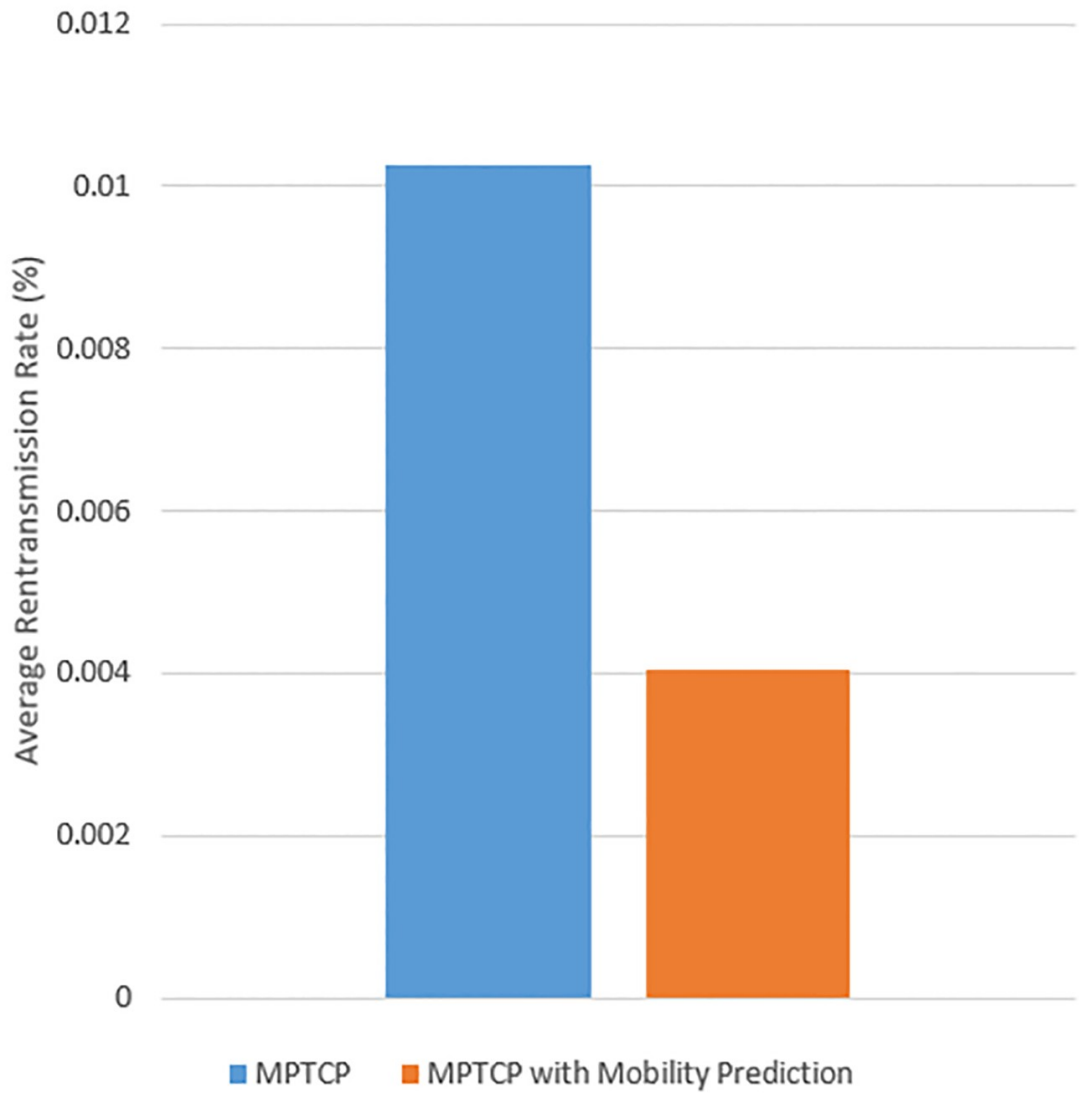
The average retransmission rate using MPTCP and MPTCP with mobility prediction.

Fig 28 shows the comparison of the average retransmission rate between MPTCP only and MPTCP with mobility prediction. The results shows that MPTCP with mobility prediction incurs a lower retransmission rate at 0.004% and that MPTCP has a retransmission rate of 0.01%. The reason is that MPTCP with mobility prediction assists the mobile device to connect to the next WiFi AP in a faster manner, thus allowing the mobile device to communicate with the server without error. However, MPTCP only connects to the random surrounding WiFi AP when the connected WiFi AP breaks, which leads to poor network connectivity, thus causing the server’s inability to receive an acknowledgement message from the mobile device. As such, the server retransmits TCP until the mobile device successfully replies with an acknowledgement message to the server.

Even though MPTCP allows mobile devices to use both WiFi and the cellular interface in the downloading activity, the selection of the WiFi AP plays an important role to maintain a good network performance for the user experience. Thus, using MPTCP without mobility prediction can cause a mobile device to continue to associate with the poor signal of the WiFi AP instead of employing the handover to a better performing WiFi AP. eHMP performs the heterogeneous handover from WiFi to cellular by optimizing the network interface utilization. This is different from MPTCP with mobility prediction that uses the multipath TCP after predicting the optimal WiFi AP.

**Fig 29:**
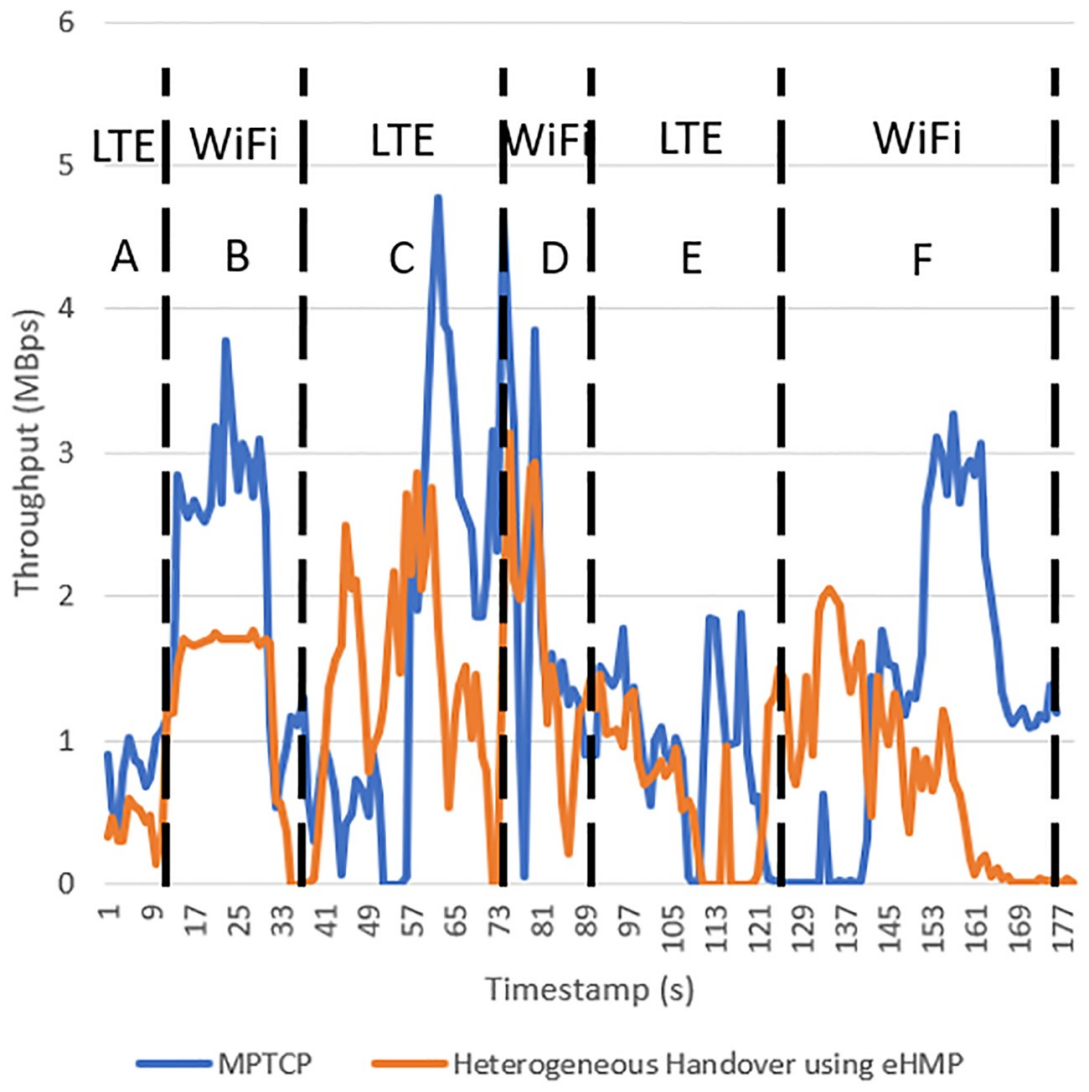
Network throughput of MPTCP and heterogeneous handover using eHMP.

Fig 29 compares the network throughput of MPTCP and eHMP in the heterogeneous network, and Table 3 compares the average network throughput of MPTCP and eHMP. eHMP uses the LTE cellular network path during handover. After handover is performed, LTE will no longer be used in downloading to preserve the network quota. This method ensures that the network offloads to the WiFi interface instead of MPTCP defaulting to the WiFi and LTE mode, where both interfaces will be used based on the round trip time and congestion window. In phase A as shown in Fig 29, the mobile device with eHMP is using the cellular network path as the primary path for downloading compared to MPTCP, which uses WiFi and cellular paths according to the RTT and the congestion window. Only at phase B, where the mobile device is connected to the predicted optimal WiFi AP, will the WiFi network path be used as the primary path for downloading. Similarly, the mobile device performs the handover process to the predicted optimal WiFi AP at phase C; thus, the cellular path is used as the primary path for the downloading activity. Only when the mobile device is successfully connected to the optimal WiFi AP will the WiFi network path be used as the primary path at phase D. Similarly, the mobile device uses the cellular path as the primary path upon the detected handover to the optimal WiFi AP that is required at phase E. The mobile device continues to use WiFi after successful handover.

**Table 3:** Average throughput of MPTCP and heterogeneous handover using eHMP in each range.

Range	MPTCP (MBps)	Heterogeneous Handover using eHMP
A	0.4063	0.7730
B	1.3326	2.2102
C	1.3564	1.5865
D	1.6835	1.9356
E	0.6838	0.9288
F	0.7740	1.3005

**Fig 30:**
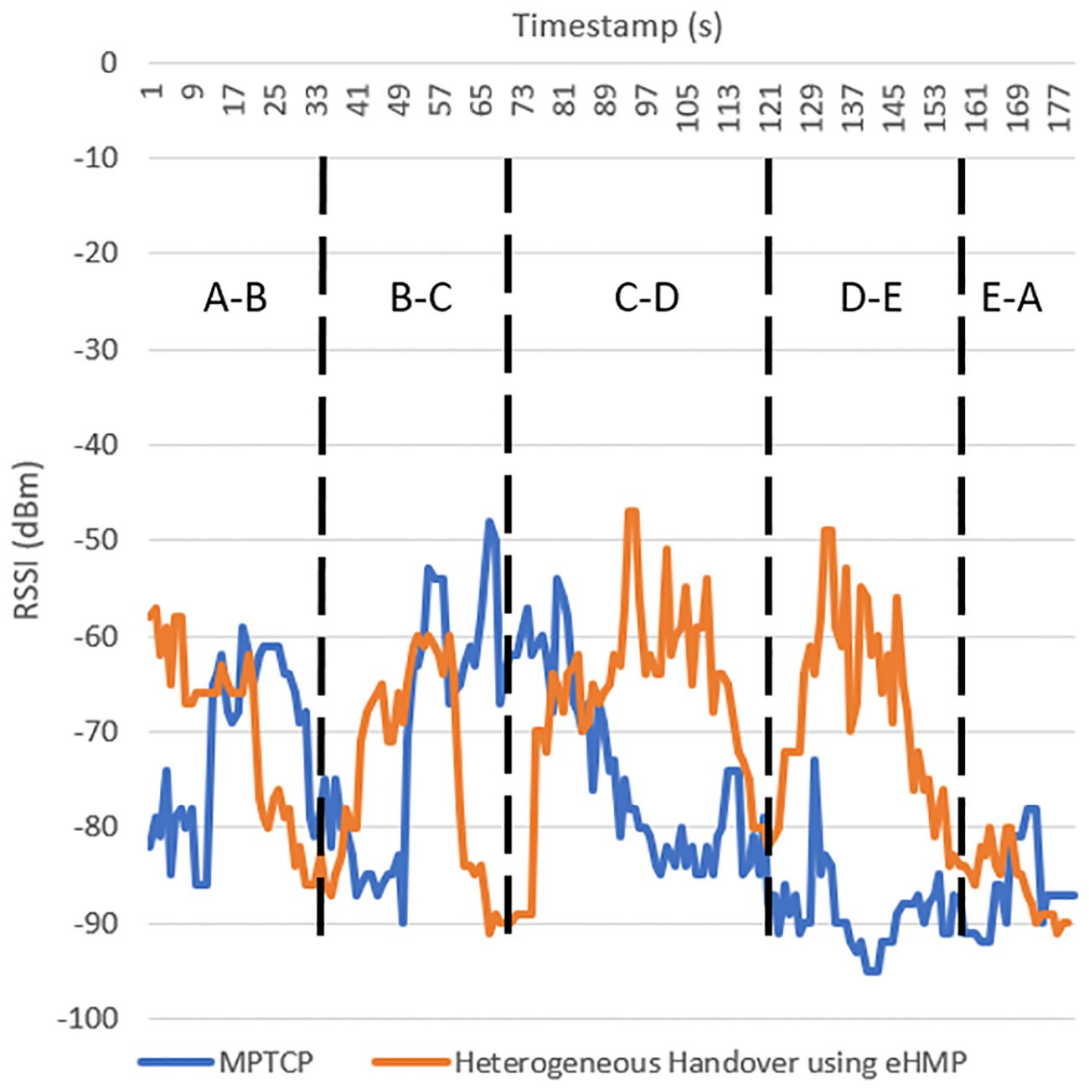
RSSI of connected WiFi AP in MPTCP and heterogeneous handover using eHMP.

Fig 30 shows the RSSI of the connected WiFi AP in MPTCP and eHMP. As compared to the existing MPTCP architecture, eHMP yields a higher RSSI at the beginning of the timestamp between A and B and then a lower RSSI near the end of A-B. This result may be because the mobile device that uses the proposed architecture is moving away from the connected WiFi AP, whereas the mobile device with MPTCP is connected to the closer WiFi AP at the middle of A-B. Nevertheless, the mobile device with the proposed architecture shows an improved RSSI between B and C due to the connection to the predicted WiFi AP, compared to MPTCP, which is still connected to the previous WiFi AP. Between B and C, the mobile device with MPTCP handover to the neighbouring WiFi AP shows a higher RSSI. Similarly, the proposed architecture assisted the mobile device to connect to the optimal WiFi AP during C-D, which shows a high RSSI compared to that with MPTCP. Meanwhile, the mobile device with MPTCP fails to connect to the random surrounding WiFi AP; therefore, the RSSI perceived by the mobile device drops between C-D and D-E. Alternately, eHMP predicted the optimal WiFi to the mobile device to connect to during D-E, where the RSSI perceived by the mobile device is high. Between E and A, MPTCP and eHMP show a low RSSI, which is due to the blind spot of the WiFi AP; thus, no optimal WiFi or neighbouring WiFi can provide a connection.

**Fig 31:**
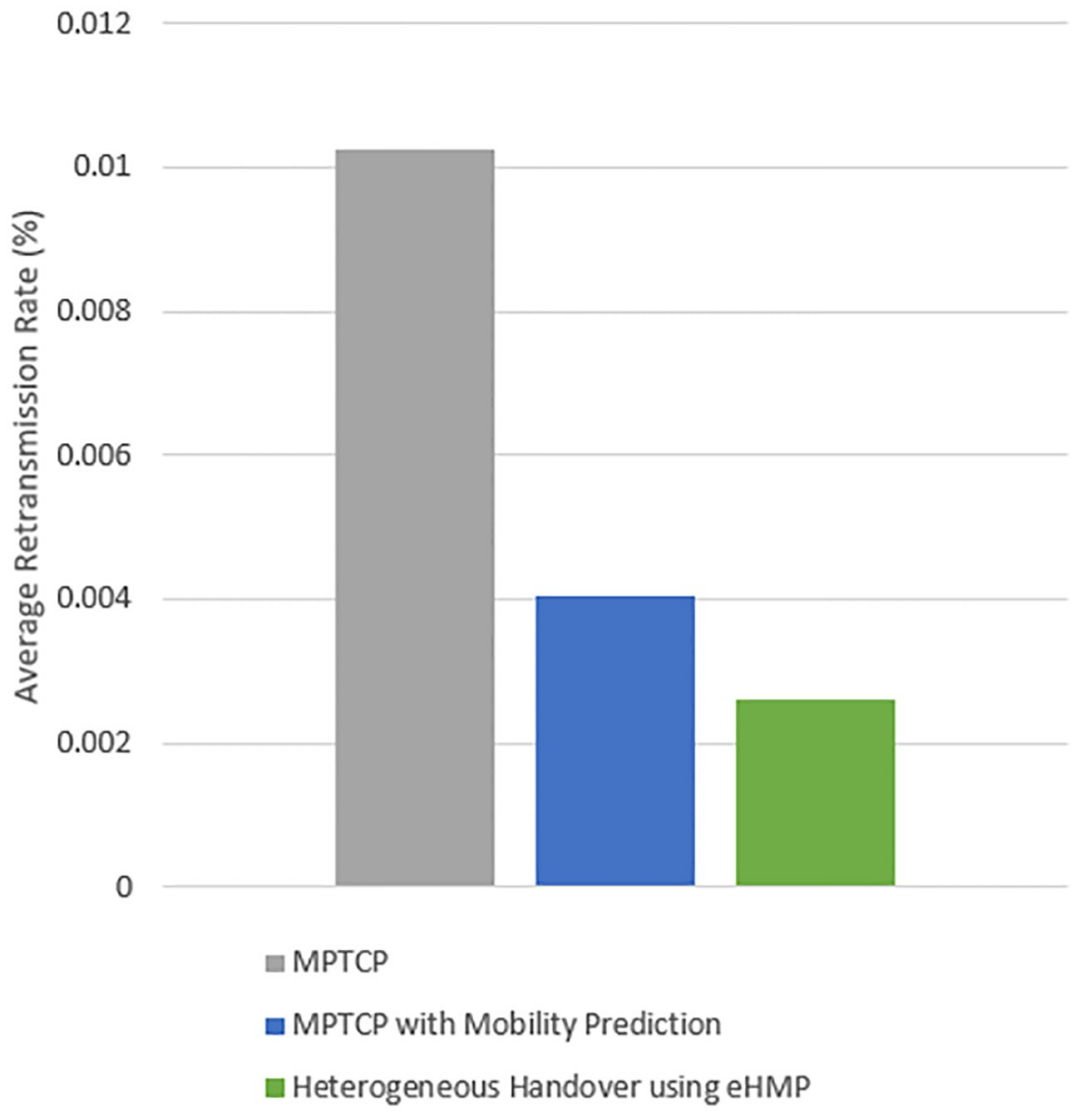
The average retransmission rate using the proposed eHMP, MPTCP and MPTCP with mobility prediction.

Fig 31 displays the average retransmission rate for the experiment using the proposed eHMP, MPTCP with mobility prediction and MPTCP. The results show that using the proposed eHMP has the lowest retransmission rate of 0.0026%, equivalent to an improvement of approximately 75% in reducing the retransmission rate of MPTCP. Using MPTCP with both WiFi and cellular interfaces has the highest retransmission rate, at 0.01%, which shows that using MPTCP without any optimization can induce extra network costs to the mobile user. Furthermore, the proposed MPTCP with mobility prediction successfully reduces the retransmission rate of MPTCP by 60.6%.

## Conclusion

This paper showed that the mobility prediction in eHMP contributed to the enhanced network performance in terms of network throughput as well as a decrease in the rate of retransmission. In the homogeneous network, eHMP increased the network throughput for TCP traffic by 106% and decreased the retransmission rate by 85%. These results show the benefit of using eHMP in mobile devices. In the heterogeneous network, an increase in network throughput by 21% was recorded for MPTCP with mobility prediction and by 55% in the heterogeneous handover with eHMP. The retransmission rate was also reduced by 40% when MPTCP with mobility prediction was used and by 75% when the heterogeneous handover with eHMP was used. The multipath mechanism that is implemented in eHMP allows mobile devices to perform the heterogeneous handover without facing any network disruption during the handover process.
